# Global burden of ischemic heart disease due to omega-3 deficiency: 204-country analysis, 1990–2021

**DOI:** 10.3389/fnut.2025.1658775

**Published:** 2025-11-26

**Authors:** Xiaozhi Chen, Lei Chen, Xinyue Yang, Zhiqiang Zhang

**Affiliations:** 1 Department of Cardiology, National Cardiovascular Disease Regional Center for Anhui, The First Affiliated Hospital of Anhui Medical University, Hefei, Anhui, China; 2 Department of Cardiology, Fujian Medical University Union Hospital, Fuzhou, Fujian, China; 3 Department of Cardiology, Beijing Chaoyang Hospital, Capital Medical University, Beijing, China; 4 Department of Cardiology, Tianjin Medical University General Hospital, Tianjin Medical University, Tianjin, China; 5 Graduate School, Tianjin Medical University, Tianjin, China

**Keywords:** ischemic heart disease, Global Burden of Disease Study, diet low in seafood omega-3 fatty acids, socio-demographic index, disability-adjusted life years (DALYs)

## Abstract

**Aim:**

This study evaluates the global burden, trends, and dietary risk factors of ischemic heart disease (IHD) from 1990 to 2021, focusing on socioeconomic and demographic variations.

**Methods:**

The study analyzed IHD-related disability-adjusted life years (DALYs), age-standardized rates (ASR), age-standardized mortality rates (ASMR), and fatality counts. Temporal trends were assessed using estimated annual percentage changes (EAPCs). Dietary risk factors, such as seafood omega-3 fatty acid deficiency, were evaluated in relation to the socio-demographic index (SDI).

**Results:**

From 1990 to 2021, DALY rates and fatalities from IHD increased globally. Omega-3 fatty acid deficiencies were identified as a significant contributor. ASMR and age-standardized death rates (ASDR) rose most notably in Central Asia and Eastern Europe. Countries with lower SDI levels faced a higher IHD burden. National trends varied, with adverse correlations between IHD burden and SDI primarily linked to dietary risks.

**Conclusion:**

Deficiencies in omega-3 fatty acids and other dietary risks are key factors driving global IHD patterns. Public health strategies to improve diet, particularly in low- and middle-SDI regions, are essential to reducing the IHD burden.

## 1 Introduction

Ischemic heart disease (IHD), characterized by left ventricular dysfunction—either systolic or diastolic—due to obstructive coronary artery disease (CAD), is a leading cause of premature death worldwide (1). GBD study conceptualizes IHD as an aggregate of discrete sequelae, encompassing myocardial infarction (MI), stable angina (manifesting as chest pain), and ischemic cardiomyopathy (heart failure resulting from IHD) (2, 3). This condition imposes substantial healthcare burdens at individual, national, and global levels. According to the American Heart Association, direct healthcare costs for cardiovascular disease increased from $103.5 billion (1996) to $251.4 billion (2018–2021) (4). Lifestyle factors including obesity, smoking, physical inactivity, and poor dietary habits increase the risk of developing IHD (5–7). Insufficient dietary intake of seafood-derived omega-3 fatty acids represents a significant risk factor. Omega-3 fatty acids, particularly eicosapentaenoic acid (EPA) and docosahexaenoic acid (DHA), are essential polyunsaturated fatty acids with unique physiological functions (8). EPA primarily exerts anti-inflammatory effects and improves blood lipid profiles, while DHA plays a critical role in myocardial cell membrane function and cardiac rhythm stability (9). Studies have shown that daily intake of EPA and DHA can significantly reduce the risk of cardiovascular diseases (10, 11). These marine-derived omega-3 fatty acids provide cardiovascular protection through multiple mechanisms, including: lowering blood cholesterol levels, reducing vascular inflammation, enhancing plaque stability, improving endothelial function, and maintaining cardiac cell membrane function (9, 12).

This recommended dose can be achieved by regularly consuming omega-3-rich deep-sea fish, such as mackerel, salmon, and tuna (13). However, global disparities in seafood consumption have resulted in inadequate omega-3 intake in many regions, contributing to an increased risk of cardiovascular diseases in affected populations (14).

Research demonstrates that consumption of seafood-derived omega-3 fatty acids reduces blood cholesterol levels while providing multiple cardiovascular benefits, including decreased inflammation, enhanced plaque stability, and improved cellular membrane function, thereby reducing IHD incidence (15). Over the past five decades, increasing seafood omega-3 fatty acid intake has emerged as a crucial strategy in preventing chronic cardiovascular disease (16, 17).

Existing studies, often limited to specific countries or regions and short time spans, fail to fully capture the long-term impact of dietary seafood omega-3 deficiency on the global burden of IHD (18, 19). This study analyzed recent GBD data to examine the global IHD burden attributable to insufficient seafood omega-3 fatty acid intake from 1990 to 2021, aiming to inform prevention strategies and dietary recommendations.

Research on gender and age stratification in IHD remains limited, with existing studies failing to fully capture the changing trends in IHD burden across different gender and age groups (20, 21). This study addresses this gap by systematically evaluating the impact of dietary omega-3 fatty acid deficiency on the IHD burden across both gender and age groups.

Previous research on the relationship between omega-3 fatty acids and IHD has primarily focused on specific countries or small populations (22, 23). In contrast, this study leverages the GBD database to comprehensively assess the effect of insufficient omega-3 fatty acid intake on IHD mortality and DALYs worldwide. By doing so, it provides a panoramic view of the global impact of this risk factor on cardiovascular health, offering valuable insights into its role in shaping IHD outcomes across diverse populations.

Beyond dietary intake, randomized trials and clinical guidance indicate preventive benefits of omega-3 supplementation for selected populations. For hypertriglyceridemia or high residual risk on statins, prescription EPA (e.g., icosapent ethyl 4 g/day) reduced total ischemic events in REDUCE-IT, while VITAL suggested benefits particularly among individuals with low baseline fish intake. Professional society statements also endorse 1–2 fish servings/week for primary prevention and higher, supervised doses for triglyceride lowering. These data contextualize our burden estimates and underscore the modifiability of omega-3–related IHD risk (10, 15, 24–26).

## 2 Materials and methods

### 2.1 Data

A database from the GBD was used in this research.^1^ The GBD 2021 incorporates the latest health surveys, including national dietary surveys, demographic data, and medical records, from an expanded number of countries and regions. These updates enhance the timeliness of the data by reflecting recent health trends and improve representativeness by capturing a broader range of populations and dietary patterns. This comprehensive integration strengthens the reliability of global health estimates and supports more accurate analyses of disease burden. From 1990 to 2021, 371 diseases and 88 risk factors from 204 countries and territories have been included in the database (27). The GBD stratifies IHD burden according to sex, age, region, and country, utilizing the socio-demographic index (SDI). The SDI integrates three metrics: fertility rates among women under 25, educational attainment in populations over 15, and per capita income distribution. The GBD framework categorizes global data into 21 distinct geographical regions, including East Asia, Oceania, and Eastern Europe. IHD prevalence was evaluated through Bayesian meta-regression analysis. Omega-3 fatty acid deficiency is defined as daily consumption below 430–470 mg of combined eicosapentaenoic acid (EPA) and docosahexaenoic acid (DHA) (28).

### 2.2 Estimation of IHD burden

The global IHD burden attributable to insufficient seafood omega-3 fatty acid intake was evaluated using mortality rates and DALYs, comprising years of life lost (YLL) and years lived with disability (YLD), from 1990 to 2021. All metrics, including ASMR and ASDR, were calculated per 100,000 population. Data were extracted using the GBD results tool.

### 2.3 Case definitions and ascertainment in GBD

IHD was harmonized using standardized ICD codes (e.g., ICD-9410–414; ICD-10 I20–I25). Cause-of-death estimates were modeled in CODEm, with redistribution of ‘garbage codes’ and covariate selection to address coding variation. Nonfatal IHD sequelae were synthesized via Bayesian meta-regression (DisMod-MR) with cross-walks for heterogeneous case definitions and adjustments for diagnostic practice changes (e.g., transition from ICD-9 to ICD-10). Verbal-autopsy inputs and surveillance data were incorporated where vital registration was sparse, improving cross-country comparability while propagating uncertainty intervals (2, 3, 28).

### 2.4 Statistical analysis

Age-standardized mortality rates (ASMR) and disability-adjusted life year rates (ASDR) were utilized to minimize demographic confounding. The EAPC formula, 100 × (exp(*β*)−1), incorporates the regression coefficient (β) derived from the linear regression of calendar year (X) (29). ASR trend classifications were determined using confidence intervals (CIs). All statistical analyses were performed using R software (version 4.3.1).

## 3 Results

### 3.1 IHD of the globally diet’s lack in omega-3 fatty acids omega-3 from seafood, 1990–2021

**Figure 1:**
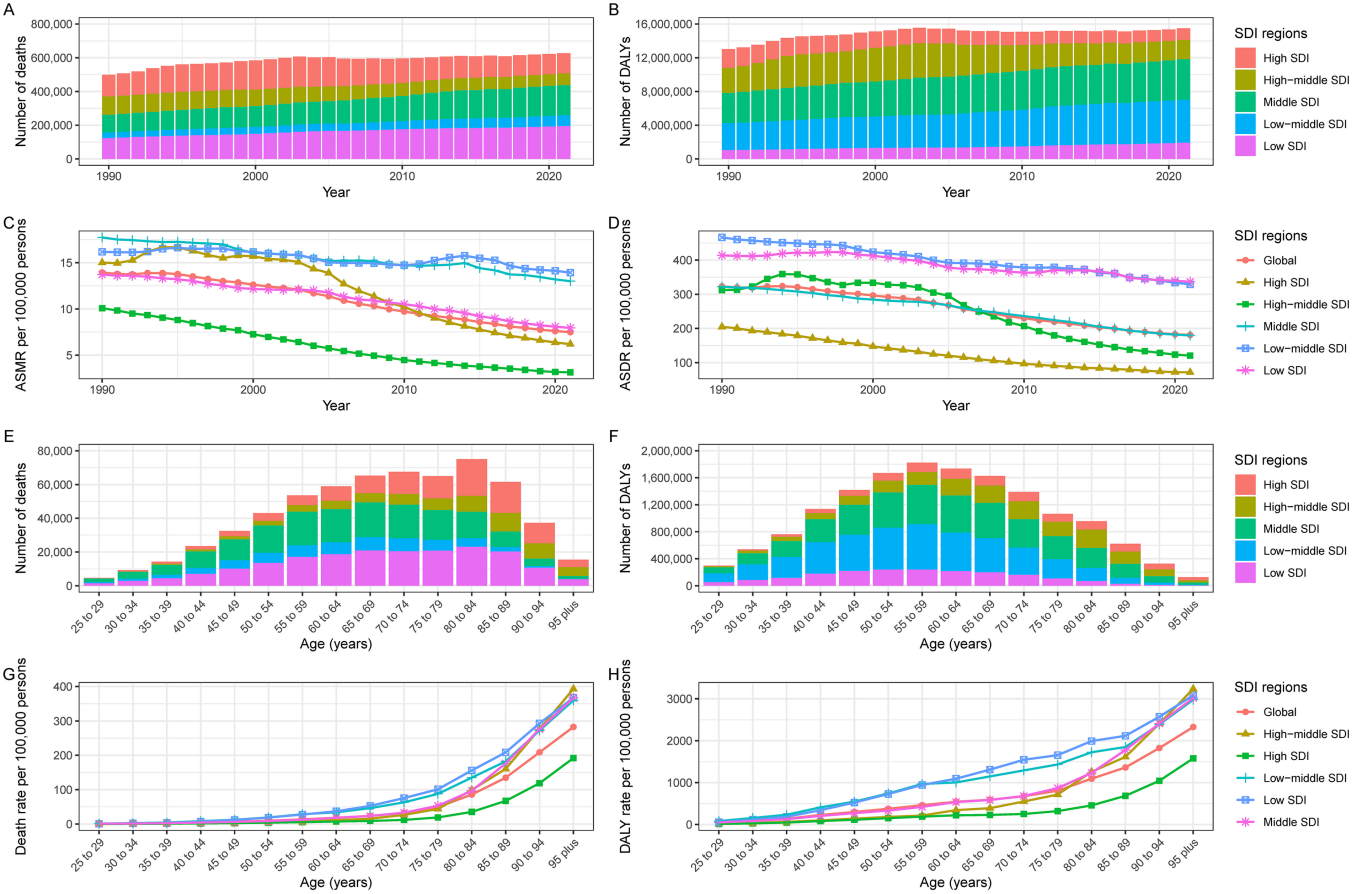
Burden of IHD due to dietary deficiencies in seafood omega-3 fatty acids in the SDI region. Global IHD **(A)** deaths, **(B)** DALYs, **(C)** ASMR, and **(D)** ASDR attributable to physical activity at all ages from 1990 through 2021. 2021 Global **(E)** Deaths, **(F)** DALYs, **(G)** Mortality, and **(H)** DALYs IHD disaggregated by lack of age.

Globally in 2021, insufficient seafood omega-3 fatty acid intake contributed to 627,300 IHD deaths (95% UI: 119,500-1,082,700) and 15,511,000 DALYs (95% UI: 3,098,800-25,946,100) (Table 1; Figures 1A,B).

**Table 1:** The global ischemic heart disease burden attributable to diet low in seafood omega-3 fatty acids in 1990 and 2021 and the temporal trends from 1990 to 2021.

characteristic	1990				2021				EAPC (1990–2021)	
	Death cases, n × 10^3^ (95% UI)	ASMR per 10^5^, n (95% UI)	DALYs, n × 10^3^ (95% UI)	ASDR per 10^5^, n (95% UI)	Death cases, n × 10^3^ (95% UI)	ASMR per 10^5^, n (95% UI)	DALYs, n × 10^3^ (95% UI)	ASDR per 10^5^, n (95% UI)	ASMR, n (95% CI)	ASDR, n (95% CI)
Global	500.2 (98.9–839.6)	13.9 (2.7–23.6)	13048.4 (2692.9–21367.2)	322.9 (65.8–533.3)	627.3 (119.5–1082.7)	7.5 (1.4–12.9)	15,511 (3098.8–25946.1)	181.1 (36.2–302.8)	−2.21 (−2.34−2.08)	−2.11 (−2.24−1.97)
Sex										
Male	254.9 (50.7–425.9)	15.4 (3–26.1)	7408.7 (1541.2–12173.8)	379 (77.2–626.5)	324.9 (61.6–559.6)	8.5 (1.6–14.7)	8,875 (1805.7–14856.3)	216.4 (43.6–363.5)	−2.12 (−2.26−1.98)	−2.05 (−2.2−1.91)
Female	245.3 (48.7–417.4)	12.4 (2.5–21.4)	5639.8 (1174.4–9,324)	266.5 (55–442.6)	302.5 (57.9–520.8)	6.5 (1.3–11.2)	6636.1 (1316.9–11123.6)	146.5 (29.2–244.7)	−2.28 (−2.4−2.16)	−2.15 (−2.27−2.04)
Socio-demographic index										
High SDI	109.6 (20.2–196.4)	10.1 (1.9–18)	2202.5 (417.1–3856.4)	204.7 (38.9–356.9)	70.2 (13–127.8)	3.1 (0.6–5.6)	1357.9 (262.6–2387.4)	71.6 (14.3–123.2)	−4.01 (−4.12−3.91)	−3.59 (−3.68−3.51)
High-middle SDI	128 (24.2–219.7)	15 (2.8–26.1)	3000.5 (589.8–5032.7)	312.3 (61–526.2)	118.5 (22.4–213)	6.2 (1.2–11.1)	2304.6 (460.3–4001.7)	120.6 (24.3–208.5)	−3.39 (−3.82−2.96)	−3.73 (−4.22−3.24)
Middle SDI	123.3 (25.2–203)	13.7 (2.7–22.9)	3565.2 (755.8–5752.1)	322.4 (66.9–527.8)	194.5 (36.8–338.1)	7.9 (1.5–13.9)	4821.6 (964.1–8042.5)	179.8 (35.4–302.4)	−1.78 (−1.91−1.65)	−1.97 (−2.09−1.85)
Low-middle SDI	104 (22–170.4)	17.7 (3.7–29.7)	3218.9 (713.7–5148.4)	466.5 (100.5–756.5)	178.3 (34.9–297.4)	13 (2.5–21.9)	5101.7 (1029.9–8327.6)	329.2 (65.5–541.5)	−0.94 (−1−0.88)	−1.09 (−1.14−1.04)
Low SDI	34.4 (7.3–57.6)	16.2 (3.3–27.5)	1040.4 (230.7–1727.7)	414.1 (89.4–691.9)	65.2 (13.5–109.2)	13.9 (2.8–23.8)	1909.7 (409.5–3115.5)	336.7 (70.3–559.7)	−0.47 (−0.57−0.37)	−0.75 (−0.84−0.66)
Region										
Andean Latin America	1.6 (0.3–2.8)	8.4 (1.6–14.8)	42.6 (9–72.2)	193.2 (39.4–327.1)	2.5 (0.5–4.4)	4.3 (0.8–7.7)	58 (11.3–100.5)	95.7 (18.4–166.2)	−2.55 (−2.84−2.26)	−2.63 (−2.92−2.35)
Australasia	2.5 (0.4–4.4)	11.1 (2–19.8)	50.3 (9.1–86.4)	219.4 (40–376.5)	1.1 (0.2–2.1)	1.8 (0.3–3.4)	17.9 (3.4–33.3)	34 (6.6–62)	−6.1 (−6.26−5.95)	−6.3 (−6.51−6.1)
Caribbean	4.1 (0.8–7)	16.8 (3.2–28.7)	102 (20.4–170.2)	388.2 (76.7–651.3)	5.2 (1–9.2)	9.6 (1.9–17)	129.4 (27.3–222.4)	242.4 (51.3–416.3)	−1.97 (−2.1−1.84)	−1.66 (−1.81−1.51)
Central Asia	18.3 (3.8–30.4)	42.7 (8.7–71.4)	447.3 (96.2–727)	947.5 (201.5–1,550)	23.1 (4.5–39.6)	32.8 (6.2–56.7)	550.6 (111.2–916.6)	678.4 (134.2–1146.6)	−1.42 (−1.82−1.03)	−1.77 (−2.22−1.32)
Central Europe	39.2 (7.6–67)	28.9 (5.6–49.5)	899.6 (182.5–1494.6)	625.3 (126.6–1041.4)	27 (4.9–47.8)	11.7 (2.2–20.6)	487.3 (90.6–846.8)	228.7 (43–393.9)	−3.39 (−3.57−3.22)	−3.76 (−3.94−3.57)
Central Latin America	9.5 (1.9–16.1)	12.6 (2.5–21.7)	246.5 (50.5–406.9)	279.8 (56.2–468.4)	21.5 (4.1–37.7)	8.9 (1.7–15.6)	493.8 (100.4–845.9)	194.7 (39.4–333.6)	−1.42 (−1.67−1.17)	−1.51 (−1.78−1.24)
Central Sub-Saharan Africa	2.2 (0.4–4)	11.4 (2.1–21.1)	62.4 (12.9–116.3)	263.9 (52.2–487.7)	4.9 (0.9–8.9)	10.6 (1.9–19.3)	142 (26.1–257)	238.9 (43.4–433)	−0.61 (−0.8−0.41)	−0.69 (−0.89−0.5)
East Asia	58.2 (12–96.9)	8.7 (1.8–14.6)	1635.3 (349–2711.9)	188 (39.4–312.2)	76.9 (13.9–140.8)	4.2 (0.8–7.7)	1,545 (287.3–2818.4)	77.7 (14.5–140)	−2.1 (−2.55−1.64)	−2.67 (−3.06−2.28)
Eastern Europe	51.9 (9.3–91.8)	21.3 (3.8–37.7)	1113.1 (212.4–1938.8)	423.9 (80.7–738.9)	48.9 (9.5–90.7)	13.9 (2.7–25.6)	924.3 (183.5–1696.7)	272.2 (54.2–495.8)	−2.42 (−3.18−1.66)	−2.65 (−3.49−1.8)
Eastern Sub-Saharan Africa	6.1 (1.3–10.4)	8.7 (1.8–14.5)	187.6 (42.4–312.5)	223.1 (48.5–377.3)	11.9 (2.5–19.7)	7.6 (1.5–12.9)	358.8 (77.4–590.3)	184.7 (38.5–306.2)	−0.65 (−0.76−0.53)	−0.88 (−0.99−0.77)
High-income Asia Pacific	0.6 (0.1–1.2)	0.3 (0.1–0.7)	12.1 (2.3–24.9)	6.3 (1.2–13.2)	0.2 (0–0.4)	0 (0–0.1)	2.2 (0.4–5.3)	0.5 (0.1–1.2)	−8.01 (−8.4−7.63)	−8 (−8.35−7.65)
High-income North America	44 (8.1–78.8)	12.3 (2.3–22)	857.5 (160.6–1491.4)	252.1 (47.6–436.9)	38.3 (7.2–69.4)	5.7 (1.1–10.2)	760.5 (148.8–1325.8)	125.9 (25–216)	−2.8 (−2.99−2.62)	−2.47 (−2.64−2.3)
North Africa and Middle East	51.4 (10.9–84.7)	33.6 (7–56.4)	1474.5 (322.5–2400.3)	804.8 (172.3–1315.4)	76.7 (15.2–131.5)	18.8 (3.6–32.7)	2061.2 (431–3462.6)	420.8 (85.1–714.2)	−2.13 (−2.2−2.05)	−2.38 (−2.45−2.3)
Oceania	0.3 (0.1–0.5)	9.1 (1.8–16.2)	8.9 (1.8–15.6)	246.8 (49.6–437.7)	0.6 (0.1–1.1)	7.8 (1.3–14.3)	19.8 (3.8–36)	211.9 (38.9–380.4)	−0.3 (−0.53−0.08)	−0.28 (−0.51−0.06)
South Asia	111 (24.5–181.6)	19.3 (4.1–32.2)	3580.4 (821.3–5736.5)	530.6 (118.6–862.2)	214.3 (42.4–359)	15.1 (2.9–25.5)	6226.5 (1284.4–10237.1)	391.1 (79.5–647.9)	−0.68 (−0.76−0.6)	−0.92 (−0.98−0.86)
Southeast Asia	18.8 (3.6–32.1)	7.8 (1.5–13.6)	576 (117.8–969.7)	198.6 (38.9–337.2)	15 (2.9–27.9)	2.5 (0.5–4.7)	422.1 (86.4–766.8)	61.4 (12.3–112.7)	−3.65 (−3.87−3.43)	−3.8 (−4−3.6)
Southern Latin America	6.6 (1.3–11.5)	15.6 (3–27.1)	149.9 (30–255)	331.2 (66.3–564.8)	4.1 (0.8–7.1)	4.6 (0.9–7.9)	84.9 (16.1–148.1)	99.7 (19.1–173.7)	−3.66 (−3.83−3.49)	−3.66 (−3.81−3.5)
Southern Sub-Saharan Africa	2.3 (0.5–3.8)	8.8 (1.9–15.5)	66.3 (15–110.1)	221.1 (48.9–370.4)	4.8 (1–8.1)	9.2 (1.8–15.9)	131.7 (28.3–218.1)	214.7 (45–360.6)	0.05 (−0.39–0.49)	−0.14 (−0.59–0.32)
Tropical Latin America	12.6 (2.5–21.3)	14.9 (2.9–25.4)	360 (75.5–592.9)	364.3 (74.1–610)	14.9 (2.9–25.9)	5.9 (1.1–10.2)	395.8 (77.9–664.1)	151.4 (29.8–254.6)	−3.05 (−3.13−−2.96)	-2.96 (−3.04−2.88)
Western Europe	51.3 (9.3–92.2)	8.8 (1.6–15.8)	975.6 (184.1–1728.9)	174.9 (33.3–307.5)	22.3 (4.1–41.2)	2 (0.4–3.6)	346.7 (66.5–623.6)	37 (7.2–65.7)	−5.03 (−5.16−4.9)	−5.19 (−5.34−5.04)
Western Sub-Saharan Africa	7.7 (1.6–13.5)	10.2 (2–18.1)	200.6 (41.8–348)	224.1 (45.8–390.9)	13.3 (2.8–23.1)	8 (1.6–14.1)	352.6 (77.9–600.7)	171.1 (36–295.4)	−1.02 (−1.22−0.81)	−1.13 (−1.35−0.91)

ASMR, age-standardized mortality rate; ASDR, age-standardized disability-adjusted life year rate; EAPC, estimated annual percentage change.

Age-standardized analysis revealed a declining IHD burden despite increasing absolute numbers (Table 1). Since 1990, the absolute burden of IHD attributable to inadequate seafood omega-3 fatty acid intake has increased. The ASMR per 100,000 decreased from 13.9 (95% UI: 2.7–23.6) in 1990 to 7.5 (95% UI: 1.4–12.9) in 2021, with an EAPC of −2.21 (95% CI: −2.34 to −2.08) (Table 1; Figure 1C). The age-standardized DALY rate (ASDR) per 100,000 declined from 322.9 (95% UI: 65.8–533.3) in 1990 to 181.1 (95% UI: 36.2–302.8) in 2021, with an EAPC of −2.11 (95% CI: −2.24 to −2.08) (Table 1; Figure 1D).

### 3.2 IHD burden by age and sex in diets deficient in omega-3 fatty acids from seafood

**Figure 2:**
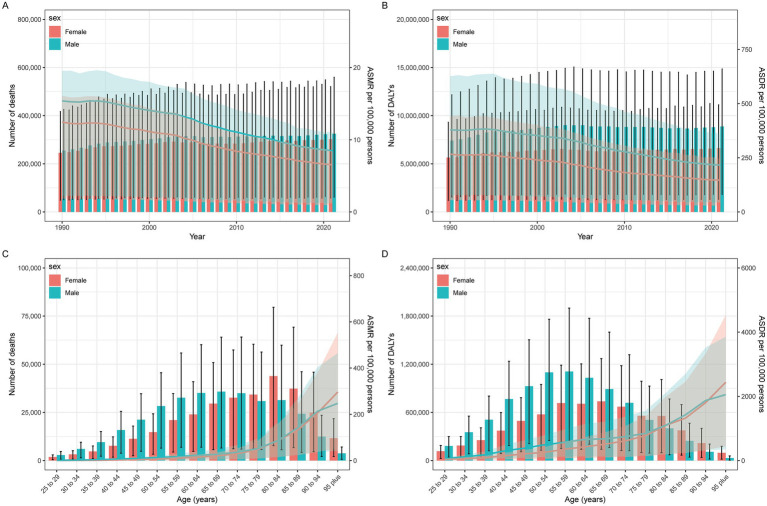
IHD year-specific **(A)** deaths and death rates and **(C)** DALY, and age-specific **(B)** deaths and death rates and **(D)** DALY for IHD by sex.

Between 1990 and 2021, IHD deaths attributable to insufficient seafood omega-3 intake increased from 254,900 (95% UI: 50.7–425.9) to 324,900 (95% UI: 61.6–559.6) in men, and from 245,300 (95% UI: 48,700-417,400) to 302,500 (95% UI: 57,900-520,800) in women. DALYs increased from 7,408,700 (95% UI: 1,541,200-12,173,800) to 8,875,000 (95% UI: 1,805,700-14,856,300) in men, and from 5,639,800 (95% UI: 1,174,400-9,324,000) to 6,636,100 (95% UI: 1,316,900-11,123,600) in women. The burden was consistently higher in men than women (Table 1; Figures 2A,B).

ASMRs decreased from 15.4/100,000 (95% UI: 3–26.1) to 8.5/100,000 (95% UI: 1.6–14.7) in men [EAPC: −2.12 (95% CI: −2.26 to −1.98)] and from 12.4/100,000 (95% UI: 2.5–21.4) to 6.5/100,000 (95% UI: 1.3–11.2) in women [EAPC: −2.28 (95% CI: −2.4 to −2.16)]. ASDRs declined from 379/100,000 (95% UI: 77.2–626.5) to 216.4/100,000 (95% UI: 43.6–363.5) in men [EAPC: −2.05 (95% CI: −2.2 to −1.91)] and from 266.5/100,000 (95% UI: 55–442.6) to 146.5/100,000 (95% UI: 29.2–244.7) in women [EAPC: −2.15 (95% CI: −2.2 to −2.04)]. Age-standardized rates showed consistent declines for both sexes (Table 1; Figures 2A,B).

Age-stratified analysis for 2021 revealed peak mortality and DALYs in the 75–79 age group, with consistently higher burdens among men (Table 1; Figures 1E,F; 2C,D).

### 3.3 By SDI region, the burden of IHD in diets lacking in omega-3 fatty acids from seafood

All five SDI quintiles showed decreasing IHD burden attributable to insufficient seafood omega-3 intake between 1990 and 2021. High SDI regions demonstrated the most substantial reductions, with EAPCs of −4.01 (95% CI: −4.12 to −3.91) for ASMR and −3.59 (95% CI: −3.68 to −3.51) for ASDR. Low SDI regions exhibited modest changes, with EAPCs of −0.47 (95% CI: −0.57 to −0.37) for ASMR and −0.75 (95% CI: −0.84 to −0.66) for ASDR (Table 1).

### 3.4 IHD burden by country and location in diets lacking in omega-3 fatty acids from seafood

**Figure 3:**
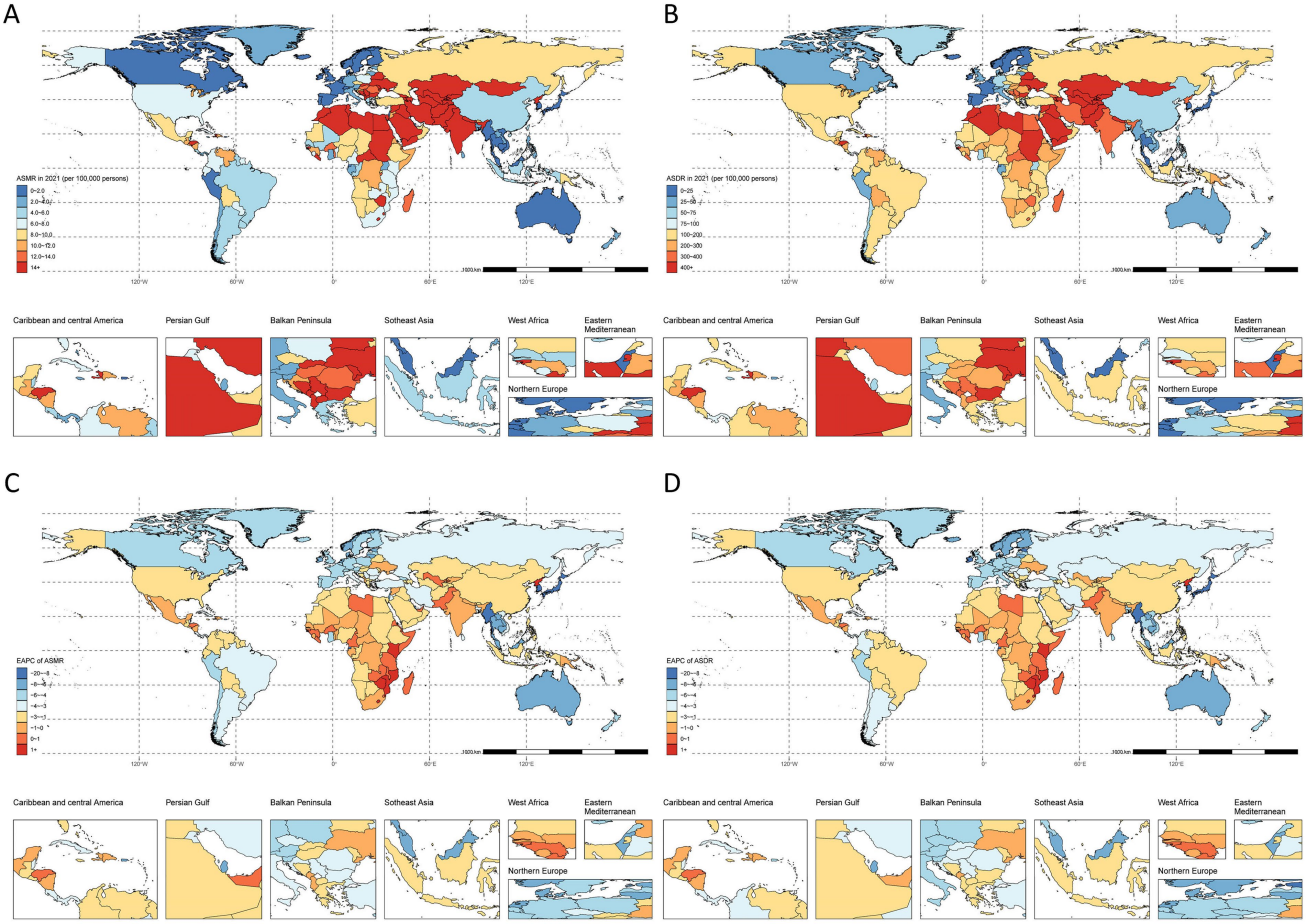
Spatial distribution of IHD due to lack of physical activity in 2021. **(A)** ASMR, **(B)** EAPC of ASMR, **(C)** ASDR, and **(D)** EAPC of ASDR.

Among 21 geographic regions in 2021, Central Asia showed the highest burden with an ASMR of 32.8/100,000 (95% UI: 6.2–56.7) and ASDR of 678.4/100,000 (95% UI: 134.2–1146.6). North Africa and Middle East followed with an ASMR of 18.8/100,000 (95% UI: 3.6–32.7) and ASDR of 420.8/100,000 (95% UI: 85.1–714.2). The lowest rates were observed in High-income Asia Pacific [ASMR: 0/100,000 (95% UI: 0–0.1); ASDR: 0.5/100,000 (95% UI: 0.1–1.2)] and Australasia [ASMR: 1.8/100,000 (95% UI: 0.3–3.4); ASDR: 34/100,000 (95% UI: 6.6–62)] (Figure 3; Supplementary Table S1).

Southern Sub-Saharan Africa uniquely showed increasing trends [ASMR EAPC: 0.05 (95% CI: −0.39 to 0.49); ASDR EAPC: −0.14 (95% CI: −0.59 to 0.32)]. Australasia and High-income Asia Pacific demonstrated the steepest declines among all other regions (Figure 3; Supplementary Table S1).

National-level analysis revealed stable trends in four countries (Côte d’Ivoire, Kiribati, Niger, Uzbekistan) and significant increases (EAPC > 2) in American Samoa, Lesotho, Nauru, and Northern Mariana Islands. The Republic of Korea showed the largest decrease (ASMR EAPC < −8). The highest death counts were observed in China [72,410,200 (95% UI: 13,118,600–133,501,900)], USA [36,886,200 (95% UI: 6,932,300–66,672,000)], and Pakistan [29,989,800 (95% UI: 6,481,200–52,583,600)]. India led in DALYs [5,066,234,400 (95% UI: 1,041,340,000–8,314,562,100)], followed by China [1,428,781,500 (95% UI: 265,229,100–2,626,045,200)] and Pakistan [942,733,100 (95% UI: 210,904,600–1,619,260,200)] (Figure 3; Supplementary Table S1).

Age-stratified analysis for 2021 showed increasing death and DALY rates with age, peaking after 80 years across all locations (Figures 1G,H; Supplementary Figures S1, S2).

### 3.5 Correlation between ASMR, ASDR and SDI values for IHD

**Figure 4:**
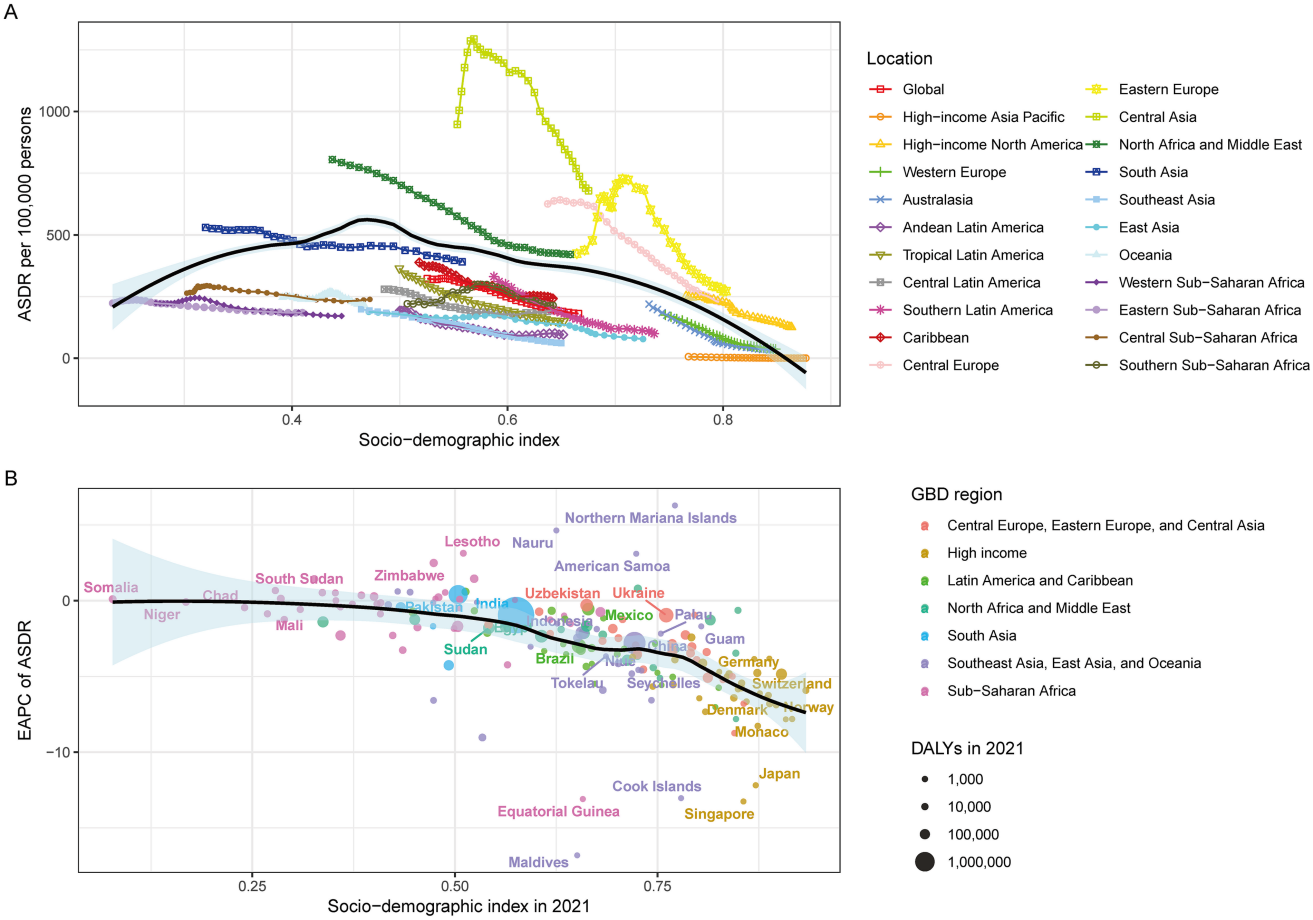
IHD in GBD areas in 2021. **(A)** Relationship between ASMR and SDI, **(B)** Relationship between Super GBD areas in ASMR and SDI in 2021.

**Figure 5:**
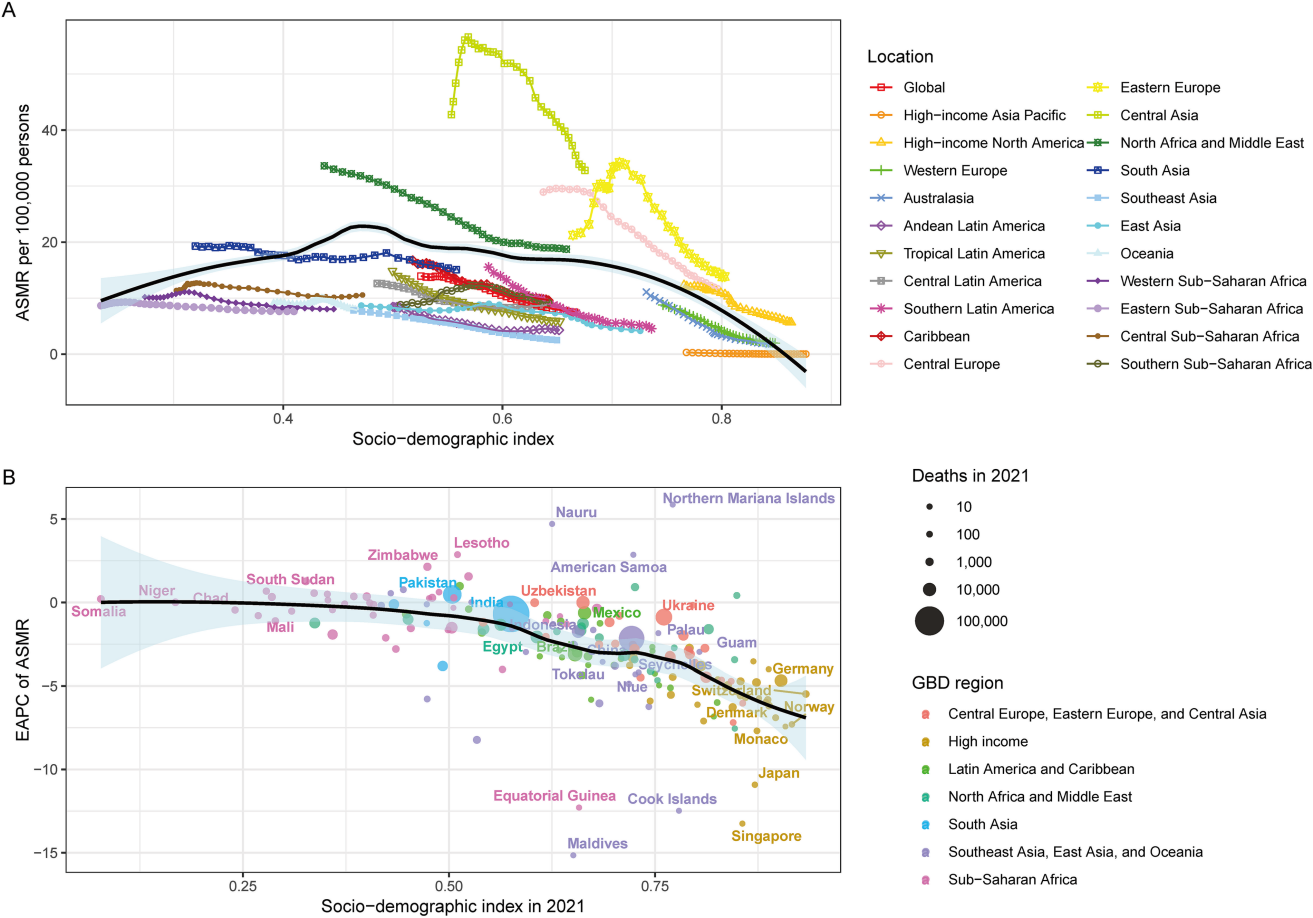
IHD in the 2021 GBD region. **(A)** Relationship between ASDR and SDI, **(B)** EAPC in the 2021 ASDR and SDI in the 2021 Super GBD region.

SDI analysis revealed a positive ASMR correlation up to SDI 0.46, followed by an inverse relationship (Figure 4A). Countries with SDI > 0.5 demonstrated negative EAPC correlations in 2021 (Figure 4B). Similar changes were observed between ASDR and SDI (Figure 5).

## 4 Discussion

From 1990 to 2021, the global IHD burden attributable to low seafood omega-3 intake showed falling ASMR/ASDR despite rising absolute deaths/DALYs; men and older adults consistently bore higher burdens; a strong SDI gradient was evident, with the steepest declines in high-SDI settings and limited progress in low-SDI regions; marked country heterogeneity included extremely low rates in high-income Asia-Pacific and unfavorable trends in selected small island states.

Omega-3 fatty acids, particularly eicosapentaenoic acid (EPA) and docosahexaenoic acid (DHA), exert cardiovascular protective effects through multiple molecular mechanisms. These include anti-inflammatory actions (e.g., suppression of pro-inflammatory factors), reduction of triglyceride synthesis, stabilization of atherosclerotic plaques, improvement of endothelial function, and anti-arrhythmic properties (24, 30). Population studies support fish consumption as a preventive measure against coronary artery disease, chronic inflammation, and metabolic syndrome (31). Epidemiological studies have demonstrated an inverse association between dietary fish consumption and coronary heart disease mortality, with optimal benefits (40–60% risk reduction) observed at daily fish intake of 40-60 g (32). Omega-3 fatty acid supplementation reduces the risk of various cardiovascular conditions, including coronary artery disease, stroke, cardiac arrhythmias, and cardiovascular mortality (30, 33, 34). Despite advances in conventional therapies such as statins and antiplatelet agents in reducing cardiovascular morbidity and mortality, additional preventive strategies remain necessary. Two large cardiovascular outcome trials of omega-3 fatty acids reached conflicting conclusions. REDUCE-IT (icosapent ethyl; pure EPA; mineral oil comparator) reported significant reductions in major adverse cardiovascular events, whereas STRENGTH (EPA + DHA carboxylic acids; corn oil comparator) was neutral and terminated for futility (25, 26, 35, 36). Potential explanations include differences in formulation/active agent (pure EPA vs. EPA + DHA), comparators and their effects on lipids and inflammatory markers, achieved EPA exposure, baseline risk, adherence, and event rates. Elevated serum omega-3 levels correlate with reduced cardiovascular event risk, supporting their role in both primary and secondary prevention strategies for high-risk populations (37–39). While some studies demonstrate associations between dietary omega-3 fatty acids and chronic disease outcomes in free-living populations (40, 41), others report no significant correlations (42, 43). These mechanisms collectively explain the outcomes observed in randomized trials, such as the reduction in ischemic events with high-dose EPA in REDUCE-IT. Thus, the benefits of omega-3 intake stem from both biological mechanisms and population-level outcome measures, like decreased cardiovascular mortality. These inconsistent findings may be attributed to geographic dietary variations, methodological limitations in dietary assessment, or differences in fatty acid bioavailability.

Two large cardiovascular outcome trials of omega-3 fatty acids reached conflicting conclusions. REDUCE-IT (icosapent ethyl; pure EPA; mineral oil comparator) reported significant reductions in major adverse cardiovascular events, whereas STRENGTH (EPA + DHA carboxylic acids; corn oil comparator) was neutral and terminated for futility (36). Potential explanations include differences in formulation/active agent (pure EPA vs. EPA + DHA), comparators and their effects on lipids and inflammatory markers, achieved EPA exposure, baseline risk, adherence, and event rates. From a dietary perspective, because seafood typically contains both EPA and DHA, STRENGTH may be compositionally more akin to habitual intake; however, pharmaceutical doses and exposures are far higher than those achieved with diet. Accordingly, these trials provide context and biological plausibility, but they should not be conflated with our GBD-based estimates of population-level burden attributable to low seafood omega-3 intake.

Studies show that men consistently have higher IHD mortality and DALY burdens than women. Men typically have higher exposure rates to cardiovascular risk factors, including obesity, smoking, and hypertension, which significantly increase the incidence and mortality of IHD (4, 20). Additionally, hormonal changes in postmenopausal women, particularly the decline in estrogen levels, also significantly increase the risk of cardiovascular diseases (44). Estrogen typically has cardioprotective effects by improving endothelial function, regulating lipid levels, and reducing inflammation, thereby lowering cardiovascular risk (45). The reduction of estrogen after menopause may lead to the loss of these protective effects, thereby increasing the risk of IHD in older women (46).

Age plays a key role. Research shows that IHD mortality and DALYs burdens peak in individuals aged 75–79, likely due to the accumulated effects of atherosclerosis, cardiovascular degeneration, and other chronic conditions in older adults (21). Cardiac structural and functional changes in older adults, including myocardial fibrosis, coronary sclerosis, and decreased vascular compliance, further elevate IHD risk (47). Moreover, older adults frequently present with comorbidities such as diabetes, chronic kidney disease, and hypertension, which commonly overlap with cardiovascular diseases, intensifying the overall IHD burden (48–51).

Men and older adults may be more sensitive to insufficient omega-3 fatty acid intake (52). Omega-3 fatty acids, particularly EPA and DHA, possess anti-inflammatory, lipid-lowering properties, enhance endothelial function, and stabilize atherosclerotic plaques (12, 53). Elevated chronic systemic inflammation in men and older adults may impair the body’s capacity to repair cardiovascular damage, thereby increasing reliance on the protective benefits of omega-3 fatty acids (49, 54). Omega-3 deficiency may further exacerbate inflammatory responses, leading to an increased risk of IHD (53, 55).

Our findings align with evidence that marine omega-3 s (EPA/DHA) exert triglyceride-lowering, anti-inflammatory, plaque-stabilizing and antiarrhythmic effects, supporting targeted supplementation strategies. In high-risk patients with residual risk on statins, purified EPA lowered ischemic event burden (REDUCE-IT), whereas population-wide guidance emphasizes regular fish consumption as a pragmatic foundation. Translating these insights to low- and middle-SDI settings requires context-specific access, affordability, and food-system policies (12, 15, 24–26).

Singapore (high-income Asia-Pacific) exhibited very low omega-3-attributable IHD rates, plausibly reflecting high SDI, strong CVD prevention programs, and comparatively high seafood access. In contrast, Nauru (small island state) showed increasing trends, consistent with its heavy cardiometabolic risk burden and small-population rate instability (wide UIs). The Maldives combines very high per-capita fish intake with small-population data constraints, yielding extreme values with broader uncertainty. GBD explicitly addresses sparse vital registration via covariates, garbage-code redistribution, and DisMod-MR synthesis; nevertheless, residual bias from diagnostic capacity, coding practice, and survey representativeness may persist, and we now acknowledge these uncertainties in the limitations.

Moreover, socioeconomic factors and lifestyle differences may further explain these gender- and age-related disparities. Additionally, socioeconomic factors and lifestyle differences may further exacerbate the disparities. In high-SDI regions, the burden of IHD has decreased significantly (EAPC for ASMR and ASDR are −4.01 and −3.59, respectively), while improvements in low-SDI regions are smaller, and some areas (e.g., parts of sub-Saharan Africa) show negative trends or remain stable. In high-SDI regions, healthcare systems and technologies are more advanced, enabling early diagnosis, treatment, and management of cardiovascular diseases. Statin medications can reduce cholesterol levels, and antiplatelet therapy can prevent thrombosis. These measures have reduced the incidence and mortality of ischemic heart disease (IHD) (56). In low-SDI regions, inadequate medical facilities and resources hinder the timely diagnosis and treatment of IHD (50). Economic development has increased per capita income, improving dietary quality (57). More people can afford to eat deep-sea fish rich in Omega-3 fatty acids, such as salmon, mackerel, and tuna, thereby reducing health risks associated with Omega-3 deficiency (14). People in low-SDI regions often lack sufficient Omega-3 fatty acid intake due to monotonous diets centered around grains and economic constraints limiting access to seafood (52). The COVID-19 pandemic disrupted food supply chains, further limiting access to nutrient-rich foods in these regions and exacerbating nutritional deficiencies (58, 59).

Projected demographic shifts indicate that by 2050, individuals aged over 60 will comprise more than 20% of the global population (60), a trend that will further increase the burden of IHD in older populations. Increasing omega-3 fatty acid intake should be prioritized as a core preventive measure, particularly among high-risk groups. Furthermore, comprehensive interventions (including smoking cessation, limiting alcohol consumption, promoting fish intake, reducing sodium intake, increasing physical activity, and improving cardiovascular care in resource-limited settings) can significantly reduce the incidence and mortality of cardiovascular diseases. Finally, further research is needed to delve into the specific mechanisms underlying gender and age differences.

This study confirms the critical role of omega-3 fatty acid deficiency in contributing to the burden of CVD, aligning with findings from existing literature (61). For example, Mozaffarian and Wu (19) highlighted that Omega-3 fatty acids reduce cardiovascular disease risk by lowering blood lipids, reducing inflammation, and stabilizing atherosclerotic plaques. This study found that the decline in IHD burden in high-SDI regions aligns with Yusuf et al. (56), who emphasized that improving healthcare infrastructure and promoting healthy diets effectively reduce cardiovascular disease burdens. Additionally, our research showed that the increasing IHD burden in low-SDI regions (e.g., sub-Saharan Africa) is associated with inadequate omega-3 fatty acid intake, consistent with Micha et al. (52), who reported dietary deficiencies in low-income countries. This study also found that men and older adults experience a higher IHD burden, supporting Virani et al. (20). They explained that men face higher IHD risks due to greater exposure to factors like smoking and hypertension, while older adults are more affected because of advanced atherosclerosis. This study specifically focuses on the effects of insufficient omega-3 fatty acid intake, whereas other dietary risk-related studies have examined the impact of high sugar, high salt, or low-fiber diets (62). By emphasizing omega-3 deficiency, this study fills a significant research gap in the global understanding of this dietary risk factor. Compared to commonly studied dietary risks in GBD 2019 literature, research on omega-3 fatty acid deficiency is more novel and holds greater academic significance (63–66).

Marine omega-3 long-chain polyunsaturated fatty acids may influence several intermediate cardiovascular risk factors. First, EPA/DHA lower triglycerides primarily by reducing hepatic VLDL production and enhancing clearance, yielding clinically meaningful reductions (67, 68). Second, small decreases in blood pressure and improvements in endothelial and inflammatory profiles have been reported (69). Third, omega-3 s exert a modest negative chronotropic effect, lowering resting heart rate—an emerging cardiovascular risk factor. Meta-analyses demonstrate that omega-3 supplementation reduces resting heart rate by approximately 1.6 beats per minute (bpm), with dose-dependent effects observed at intakes ≥1 g/day (70). This heart rate reduction is clinically meaningful, as epidemiological evidence shows that each 1-bpm increase in resting heart rate is associated with a 2% increase in all-cause mortality and a 1% increase in cardiovascular mortality (71, 72). The heart rate-lowering effect of omega-3 fatty acids may contribute to their antiarrhythmic protection and overall cardiovascular benefit. These mechanisms are consistent with—but not equivalent to—clinical endpoint reductions and provide biological plausibility for the attributable burden estimated in our study.

This study provides a valuable contribution to understanding the global burden of IHD associated with omega-3 fatty acid deficiency. Using data from the GBD 2021 database, we analyzed the impact of dietary risk factors on the global burden of IHD. The GBD 2021 database spans data from 1990 to 2021 and includes updated results for the most recent 2 years, particularly addressing the disease burden caused by the COVID-19 pandemic in 2020 and 2021, along with adjustments to the burden of other diseases. Importantly, GBD 2021 incorporates model optimizations based on GBD 2019. Spanning the years 1990 to 2021, this study illustrates the long-term trends in the IHD burden related to omega-3 deficiency, particularly quantifying regional burden changes using the EAPC metric. While previous research on the global impact of omega-3 fatty acids on IHD has been limited, this study comprehensively quantifies the disease burden of omega-3 deficiency using GBD data, along with an exploration of mechanisms and regional differences, thereby addressing this critical gap.

## 5 Limitations

There were some limitations of this study that were worth noting. This study utilizes data from the GBD database, which does not differentiate between the specific effects of DHA and EPA, making it impossible to assess their distinct impacts on the IHD burden. The GBD database integrates data from diverse sources, including national health surveys, scientific publications, and statistical models. However, data quality and completeness vary significantly across countries, with low- and middle-income regions often experiencing data gaps or inaccuracies due to underdeveloped health surveillance systems, potentially biasing global IHD estimates.

Although this study spans 32 years and covers 204 countries, it does not account for detailed temporal changes in dietary patterns, fish consumption, or seafood availability. The assumption of regional homogeneity may also obscure significant subnational variations in omega-3 intake and IHD burden. As an observational study based on secondary data, causality between omega-3 deficiency and IHD burden cannot be established. While statistical associations are observed, experimental studies or randomized controlled trials (RCTs) are required to validate these findings and quantify the direct effects of omega-3 intake on IHD risk reduction.

Across regions, viability of IHD diagnostic criteria varies with access to ECG/troponin testing, catheterization, and clinician coding practices. Transitions from ICD-9 to ICD-10, incomplete vital registration, and differential adoption of high-sensitivity biomarkers may introduce ascertainment biases. Although GBD harmonizes definitions and applies garbage-code redistribution and cross-walks, residual heterogeneity likely remains, particularly in low-resource settings; hence, our country-level estimates should be interpreted alongside UIs and health-system context (2, 3, 28).

The estimation of ASMR and ASDR depends on assumptions within the GBD statistical models, such as using SDI as a proxy for development. These assumptions may fail to fully capture the intricate relationships between dietary practices, socioeconomic conditions, and healthcare systems, potentially limiting the precision of the results. This study specifically examines the association between omega-3 deficiency and IHD burden using GBD data, while other aspects, such as environmental influences on omega-3 levels or its role in specific subpopulations, fall outside the scope of this analysis.

As an ecological analysis, this study cannot establish causality between omega-3 deficiency and IHD outcomes. The observed associations are susceptible to confounding by unmeasured dietary factors (protein intake, micronutrients, overall dietary quality) and socioeconomic determinants beyond SDI (healthcare access, lifestyle behaviors, environmental exposures). Populations with adequate omega-3 intake typically exhibit better overall nutritional status and healthcare infrastructure. Consequently, the GBD burden estimates likely overestimate the true causal effect of omega-3 deficiency and should be interpreted as hypothesis-generating associations rather than evidence supporting single-nutrient interventions.

## 6 Conclusion

From 1990 to 2021, the IHD burden attributable to low seafood omega-3 intake declined in age-standardized terms but rose in absolute numbers, with men, older adults, and low- to middle-SDI regions most affected. These findings reinforce omega-3 intake as a modifiable factor and highlight the need for context-appropriate policies that expand access to fish or effective supplementation, particularly in resource-limited settings. Strengthening diagnostic capacity and data systems will improve comparability and targeting of prevention.

## Data Availability

The original contributions presented in the study are included in the article/Supplementary material, further inquiries can be directed to the corresponding authors.

## Footnotes

^1^ http://ghdx.healthdata.org/gbd-results-tool
