# Supplementary material for: Global burden of ischemic heart disease due to omega-3 deficiency: 204-country analysis, 1990–2021
Source: Front Nutr. 2025 Nov 26;12:1658775. doi: 10.3389/fnut.2025.1658775 (PMC12689341; doi:10.3389/fnut.2025.1658775)
Supplement: Supplementary file 1 [file Supplementary_file_1.docx]

# Supplementary Figure legends


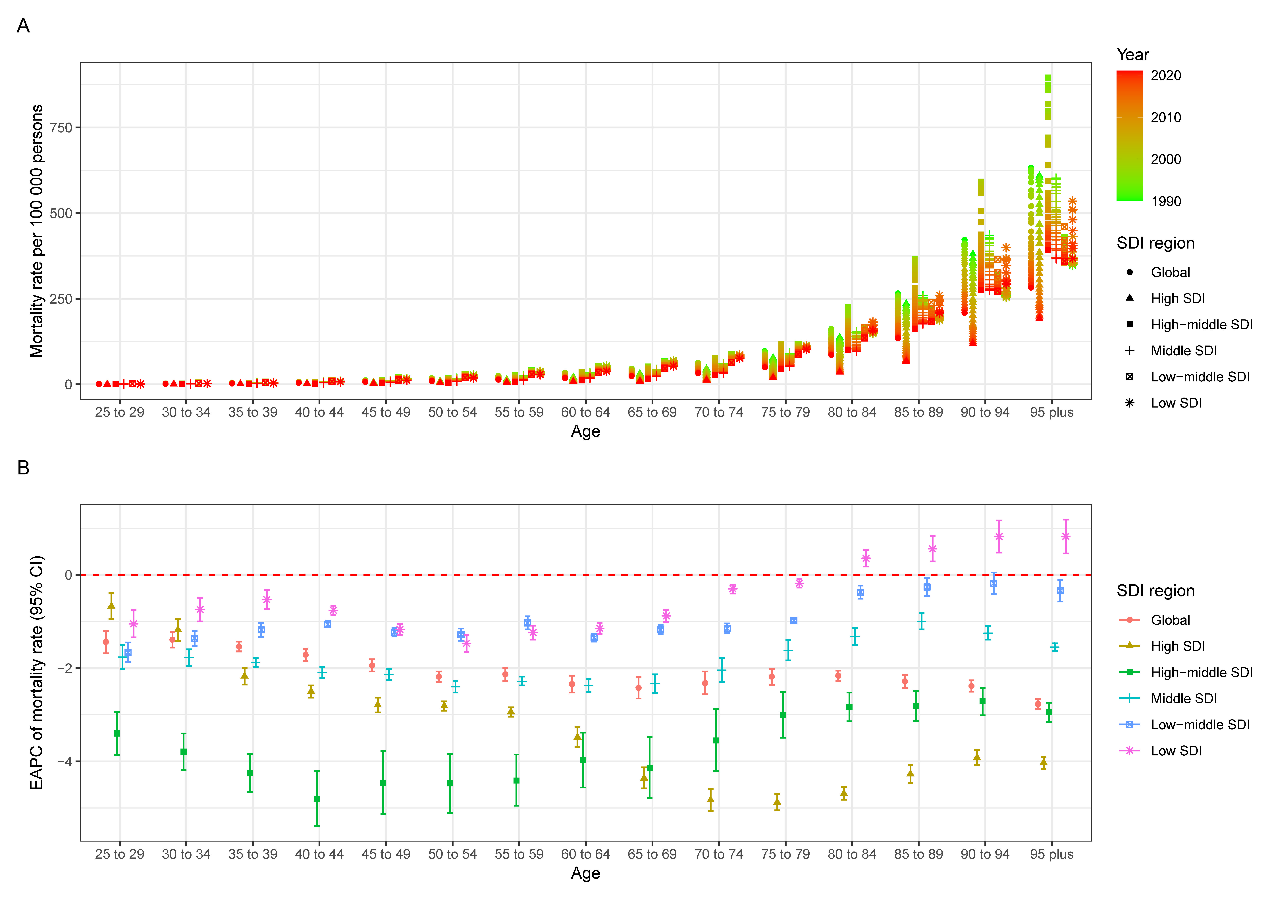


Figure S1: The age distribution of (A) age-specific mortality rate and (B) EAPC in age-specific mortality rate attributable to diet low in seafood omega-3 fatty acids by SDI region from 1990 to 2021.


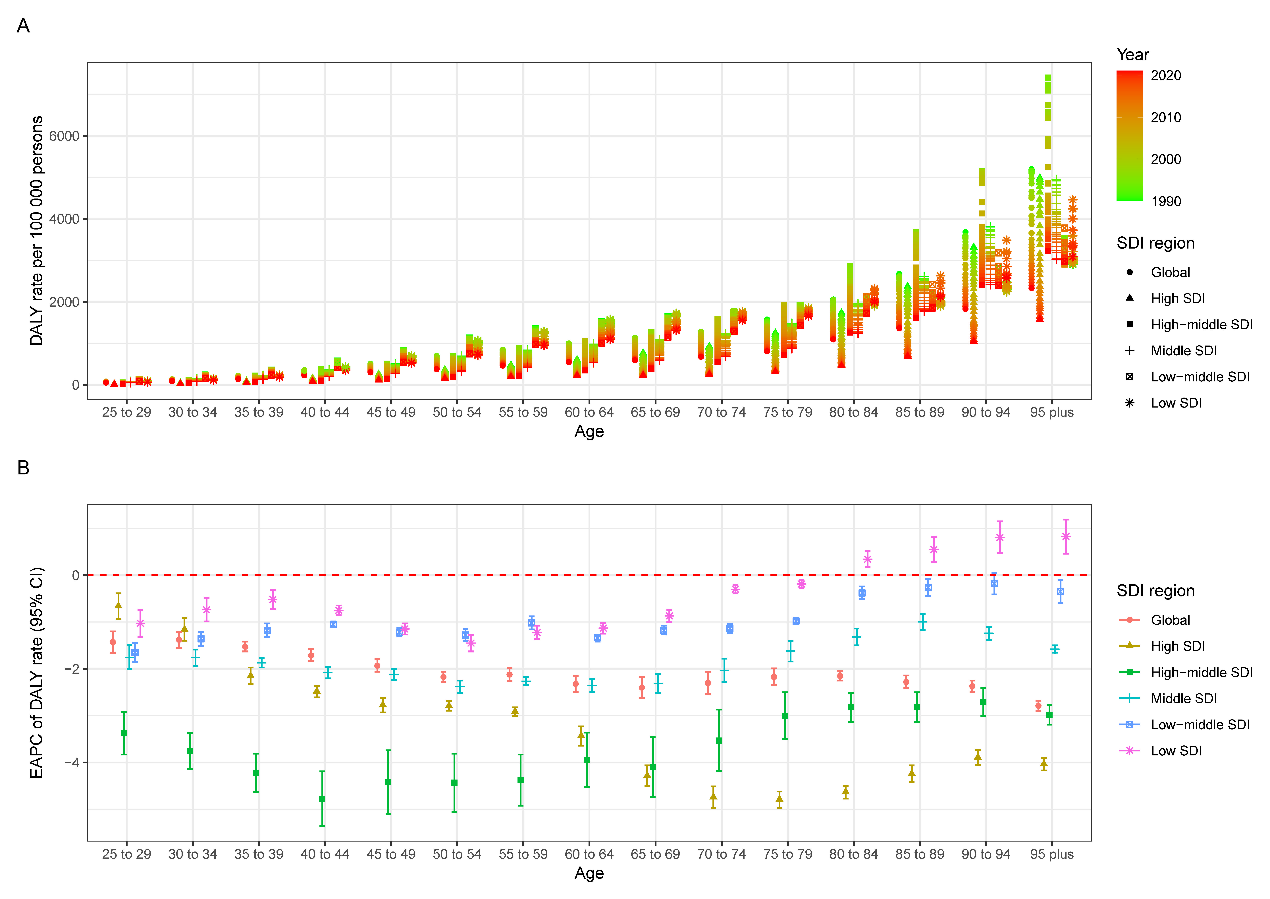


Supplementary Figure 2: The age distribution of (A) age-specific DALYs rate and (B) EAPC in age-specific DALYs rate attributable to diet low in seafood omega-3 fatty acids by SDI region from 1990 to 2021.

# Supplementary Tables

| **characteristic** | **1990** | | | | **2021** | | | | **EAPC (1990-2021)** | |
| --- | --- | --- | --- | --- | --- | --- | --- | --- | --- | --- |
|  | **Death cases, n (95% UI)** | **Death cases, n (95% UI)** | **Death cases, n (95% UI)** | **Death cases, n (95% UI)** | **Death cases, n (95% UI)** | **ASMR per 105, n (95% UI)** | **DALYs, n (95% UI)** | **ASDR per 105, n (95% UI)** | **ASMR, n (95% CI)** | **ASDR, n (95% CI)** |
| Afghanistan | 4129.9 (814.3-7166.2) | 62.6 (12.2-108.9) | 119343.4 (24144-204544.7) | 1655.8 (332-2846) | 4584.4 (1036.8-7833.7) | 46.3 (9.8-80.2) | 150423.6 (35066.7-255073.2) | 1178.9 (262.7-2021.1) | -1.23 (-1.37--1.09) | -1.4 (-1.55--1.24) |
| Albania | 404.8 (82-672.7) | 22.6 (4.5-38.2) | 9503.2 (2024.2-15233.6) | 462.2 (96.3-754.3) | 665.8 (123.6-1177.4) | 16.4 (3.1-29.1) | 12189.2 (2373-21006.1) | 297.2 (59-510.7) | -0.8 (-0.98--0.61) | -1.24 (-1.42--1.06) |
| Algeria | 3550.7 (734.2-6093.3) | 39.7 (7.8-68.7) | 98028.5 (21531.5-162723.6) | 823 (173.1-1393.8) | 6752.7 (1360-12023.7) | 24.7 (4.9-44.4) | 162014.6 (34863.4-282353.9) | 467.7 (97.1-819.1) | -1.67 (-1.75--1.59) | -2.16 (-2.3--2.03) |
| American Samoa | 0.1 (0-0.2) | 0.4 (0.1-1) | 2.2 (0.3-5.4) | 8.9 (1.2-22.4) | 0.4 (0.1-1) | 1.1 (0.2-2.5) | 12 (2.1-27.2) | 25.3 (4.4-58.1) | 2.86 (2.59-3.13) | 3.1 (2.83-3.38) |
| Andorra | 3.6 (0.7-6.5) | 7.1 (1.3-13.1) | 83 (16-149.5) | 148.1 (28.2-268.4) | 4.2 (0.7-7.7) | 2.4 (0.4-4.4) | 72.9 (13.4-131.3) | 46 (8.6-83.6) | -3.53 (-3.81--3.25) | -3.86 (-4.14--3.57) |
| Angola | 360.2 (76.3-632.9) | 10.7 (2.3-19) | 10742.4 (2320.7-18965.5) | 253.4 (53.8-445.1) | 824.3 (145-1521.1) | 8.5 (1.5-15.9) | 23812.8 (4148.5-42704.9) | 187.5 (33.1-346.5) | -1.54 (-1.84--1.24) | -1.76 (-2.06--1.46) |
| Antigua and Barbuda | 2.2 (0.4-4.2) | 3.9 (0.7-7.4) | 43.6 (7.9-82.9) | 81.6 (15-156.1) | 1 (0.1-1.9) | 1.1 (0.2-2.2) | 18.9 (3-38.6) | 19 (3-38.8) | -4.26 (-4.58--3.93) | -4.76 (-5.14--4.37) |
| Argentina | 5103.2 (1003.8-8838.7) | 17.1 (3.4-29.8) | 117103.2 (23561.2-198651.3) | 372.4 (75.2-632.3) | 3135.5 (596.1-5465) | 5.5 (1.1-9.6) | 65495.6 (12729.6-114485.1) | 119.6 (23.4-208.4) | -3.32 (-3.52--3.13) | -3.43 (-3.61--3.25) |
| Armenia | 995.1 (203.3-1674.3) | 41.3 (8.3-70) | 23459.7 (5006-38659.2) | 868.5 (182.6-1444.4) | 1069.3 (214.5-1846.3) | 25 (5-43.1) | 21472.9 (4447.2-36069.2) | 511.3 (106.7-852.2) | -2.48 (-2.76--2.19) | -2.48 (-2.72--2.23) |
| Australia | 2062.9 (357.2-3659.9) | 11 (1.9-19.7) | 41049 (7350.9-70172.1) | 215.1 (39-369.9) | 892.1 (160.5-1700.2) | 1.7 (0.3-3.2) | 14296.4 (2715.7-26151.6) | 32.2 (6.2-58.9) | -6.27 (-6.44--6.1) | -6.41 (-6.62--6.2) |
| Austria | 1758.1 (336.3-3117.9) | 14.5 (2.8-25.7) | 33470.6 (6467.2-57767.4) | 291.6 (56.4-500.9) | 861.1 (148.6-1625.5) | 3.8 (0.7-7.1) | 12194.2 (2181.2-22581.4) | 62.3 (11.5-113.7) | -4.72 (-4.92--4.52) | -5.44 (-5.73--5.14) |
| Azerbaijan | 2088.5 (441.7-3481.6) | 46.8 (9.7-80) | 53801.1 (11918.5-87139) | 1055.7 (229.2-1725.4) | 2921.3 (552.8-5051.4) | 34.8 (6.5-61.5) | 68864.4 (13757.8-117712.1) | 695 (134.4-1195.9) | -1.18 (-1.46--0.89) | -1.85 (-2.2--1.49) |
| Bahamas | 10.5 (2-18.8) | 7 (1.3-12.5) | 295.3 (57.9-518.5) | 174.1 (33.4-308) | 12.5 (2.4-23.5) | 3.3 (0.6-6.2) | 321.5 (64.4-610.8) | 76.7 (15.2-145.6) | -2.74 (-3.17--2.31) | -2.91 (-3.37--2.45) |
| Bahrain | 45.3 (9.3-77) | 33.3 (6.1-58.8) | 1403.8 (307.8-2335.4) | 692.2 (135.4-1193) | 59.6 (11.3-107.4) | 9.8 (1.7-18.1) | 1857.5 (370.5-3248.5) | 186.1 (34-337) | -4.66 (-5--4.32) | -5.1 (-5.43--4.77) |
| Bangladesh | 5007.2 (1013.7-8606.7) | 10.3 (2-18) | 161110.5 (34599.9-274028.9) | 293 (60.7-501.2) | 3838.9 (719.1-7511.3) | 3 (0.6-5.8) | 103944.9 (19632.5-202248.7) | 72.2 (13.5-139.9) | -3.81 (-4.05--3.57) | -4.27 (-4.45--4.09) |
| Barbados | 14.5 (2.6-28.3) | 5 (0.9-9.7) | 270.2 (49.3-512.3) | 96.8 (17.9-183.7) | 11.5 (2-21.6) | 2.2 (0.4-4.2) | 214.2 (38.5-396.9) | 43.2 (7.8-80) | -2.67 (-3.17--2.17) | -2.77 (-3.27--2.27) |
| Belarus | 3753.6 (701.9-6481.2) | 30.9 (5.8-53.1) | 78882.1 (15206.1-131789.4) | 634.8 (122.3-1060.4) | 3591.4 (589.8-6661.4) | 22.2 (3.7-41.1) | 68135.5 (12202.4-124729.8) | 438 (79.8-795.3) | -1.98 (-2.52--1.43) | -2.27 (-2.91--1.62) |
| Belgium | 1051.7 (178.1-1914.8) | 6.8 (1.2-12.4) | 19837.5 (3488.5-35949.8) | 134.9 (24.4-244.8) | 347.1 (61.2-687.1) | 1.2 (0.2-2.4) | 5487.2 (1019-10862.1) | 23.4 (4.4-46) | -5.58 (-5.72--5.45) | -5.76 (-5.88--5.63) |
| Belize | 10 (1.9-17.2) | 10.7 (2-18.6) | 239.8 (47.1-401.4) | 249.6 (48.5-420.5) | 13 (2.4-23.1) | 4.6 (0.8-8.3) | 335.2 (66.8-572.6) | 105.3 (20.5-184.5) | -3.23 (-3.53--2.93) | -3.32 (-3.6--3.04) |
| Benin | 118.7 (24.1-216.2) | 6.5 (1.3-11.9) | 2968.9 (641.1-5220.4) | 146.2 (30.9-260.3) | 283.8 (53.3-510.4) | 6.3 (1.2-11.6) | 7468.4 (1488.1-13160.4) | 137.3 (26.2-244.8) | -0.09 (-0.25-0.07) | -0.23 (-0.41--0.05) |
| Bermuda | 4.5 (0.8-8.5) | 8 (1.5-15) | 96.2 (18.8-180.6) | 156.9 (30.2-292.3) | 1.7 (0.3-3.6) | 1.1 (0.2-2.4) | 28.3 (4.5-58.2) | 21.1 (3.4-43) | -6.83 (-7.4--6.26) | -7.02 (-7.63--6.4) |
| Bhutan | 37.5 (7.9-63.7) | 15.7 (3.2-27.6) | 1223.6 (270.2-2090.3) | 419.4 (89.6-708.6) | 62.8 (11.3-111.7) | 10.7 (1.9-19.2) | 1636.7 (320.5-2846.2) | 254.4 (48.9-446.1) | -1.24 (-1.28--1.2) | -1.69 (-1.74--1.64) |
| Bolivia (Plurinational State of) | 558.8 (101.3-1015.2) | 19.6 (3.4-35.4) | 15164.7 (2972.3-27457.8) | 446.7 (83.7-806.1) | 789.8 (139.9-1488.5) | 9.9 (1.7-18.7) | 19443.4 (3565.2-35551.8) | 211.4 (38.4-391.4) | -2.18 (-2.4--1.96) | -2.48 (-2.72--2.23) |
| Bosnia and Herzegovina | 1046.5 (217-1749.1) | 28.7 (5.8-48.6) | 26917.5 (5860.8-43817.9) | 644.8 (137.5-1064.9) | 969.7 (183.6-1749.4) | 15.5 (3-27.9) | 18434 (3491.3-32816.1) | 307 (59.4-545.4) | -2.54 (-2.87--2.21) | -2.95 (-3.25--2.66) |
| Botswana | 68.9 (13.9-119.2) | 14.5 (2.7-25.3) | 1980.3 (426.5-3416.9) | 339.3 (69.5-584.8) | 126.5 (27.5-220.4) | 9.9 (2.1-17.7) | 3554.1 (802.7-6142.1) | 223.8 (48.7-391.1) | -1.3 (-1.59--1) | -1.52 (-1.86--1.18) |
| Brazil | 12393 (2455-20884.8) | 14.9 (2.9-25.6) | 353621.7 (74191.3-582784.9) | 366.5 (74.6-613.8) | 14488.9 (2807.3-25225.1) | 5.8 (1.1-10.1) | 385223.5 (75917.9-646961.2) | 150.9 (29.7-254.1) | -3.09 (-3.18--3.01) | -3 (-3.08--2.92) |
| Brunei Darussalam | 3.1 (0.6-6.2) | 3.3 (0.6-6.5) | 93.5 (19.7-182.5) | 70.6 (14.7-142.3) | 6.7 (1.2-13.1) | 2.4 (0.4-4.7) | 203.9 (41.2-388.1) | 51.6 (9.6-99.1) | -1.32 (-1.65--0.98) | -1.49 (-1.88--1.11) |
| Bulgaria | 3799.5 (758.1-6574.7) | 42.8 (8.3-74.4) | 85863.5 (17628.2-144658.8) | 810.2 (165-1368.2) | 2743.8 (519.8-4769.1) | 20.3 (3.9-34.9) | 54596.4 (10836-92668.3) | 437.1 (90.4-741.6) | -3.19 (-3.59--2.78) | -2.84 (-3.25--2.44) |
| Burkina Faso | 451.4 (94.7-780.7) | 12 (2.4-20.8) | 11768.7 (2609.6-20065.2) | 269.3 (58.1-458.9) | 991.9 (183.7-1762) | 12.4 (2.3-22.3) | 25051.8 (4845.2-43849.7) | 266.5 (49.7-464.2) | 0.33 (0.2-0.46) | 0.13 (0.02-0.23) |
| Burundi | 323.5 (66.8-563.5) | 14.7 (2.9-25.6) | 9447.3 (2095.1-16198.8) | 373.5 (80-642.7) | 558.2 (110.5-963.3) | 12.3 (2.3-21.8) | 17242.5 (3663.9-29408.5) | 300.8 (59.6-521.7) | -1.11 (-1.3--0.91) | -1.26 (-1.46--1.05) |
| Cabo Verde | 15.4 (2.9-27.7) | 6.6 (1.3-11.8) | 339.1 (67.7-597.3) | 153.4 (31.2-268.4) | 29.1 (5.2-55.6) | 6.9 (1.2-13.1) | 621.5 (115.2-1177.1) | 137.2 (24.7-262.6) | -0.64 (-1.04--0.24) | -1.07 (-1.41--0.73) |
| Cambodia | 304.3 (59.5-534.3) | 7.3 (1.4-13.5) | 9419.6 (1905.4-16074.1) | 184.9 (36.6-322.5) | 134.5 (25.3-292.9) | 1.4 (0.3-3.1) | 3483 (669.7-7462.2) | 28.7 (5.4-62.4) | -5.78 (-6.09--5.46) | -6.58 (-6.94--6.22) |
| Cameroon | 259.9 (51.5-466.9) | 6.9 (1.3-12.4) | 6930.9 (1399.4-12370.7) | 151.9 (30.2-270) | 824.9 (172.3-1528.7) | 7.7 (1.6-14.3) | 22758.4 (4868.1-41810.4) | 168.9 (35.4-313.1) | 0.28 (-0.32-0.89) | 0.22 (-0.42-0.87) |
| Canada | 2198.7 (388.6-4161.2) | 6.9 (1.2-13.2) | 42591.3 (7655.4-78332.3) | 133.8 (24.1-244.3) | 1400.4 (251.5-2724.7) | 1.7 (0.3-3.4) | 23200.1 (4389.2-44419.9) | 33.2 (6.5-62.8) | -4.79 (-4.97--4.61) | -4.77 (-4.95--4.6) |
| Central African Republic | 176.4 (34.4-324) | 17.6 (3.3-32.2) | 5314.8 (1072.4-9674.8) | 426.8 (82.6-777.8) | 315.6 (64.5-577.5) | 16.1 (3.1-29) | 9963.3 (2041.4-18372.4) | 389.2 (78.6-707.5) | -0.52 (-0.63--0.41) | -0.58 (-0.69--0.46) |
| Chad | 243.2 (51.2-438.9) | 9.5 (1.9-17.2) | 6081.7 (1355-10796.3) | 214.2 (46.8-382.7) | 460.8 (98-805.1) | 9.1 (1.8-15.9) | 12773.5 (2813.1-22495.8) | 206.4 (44.1-359.4) | -0.45 (-0.75--0.15) | -0.46 (-0.79--0.13) |
| Chile | 837.6 (157.9-1471.6) | 9.5 (1.8-16.8) | 17980.6 (3519.7-31248.6) | 183.5 (35.4-319) | 573.4 (103.1-1042.6) | 2.2 (0.4-4) | 12701 (2442.5-22625) | 51 (9.9-90.2) | -4.46 (-4.58--4.35) | -3.9 (-4.01--3.79) |
| China | 56638 (11739.2-94407.3) | 8.8 (1.8-14.8) | 1592558.3 (340659.8-2656747.5) | 190.5 (40-315.6) | 72410.2 (13118.6-133501.9) | 4.1 (0.8-7.6) | 1428781.5 (265229.1-2626045.2) | 74.9 (13.9-135.9) | -2.17 (-2.64--1.69) | -2.8 (-3.21--2.39) |
| Colombia | 2825 (567.2-4689.5) | 17.2 (3.4-29) | 74786.3 (15776.1-122149.6) | 397.9 (81.8-654.9) | 4168.2 (740-7431.3) | 7.4 (1.3-13.1) | 87011.3 (16137.5-150723.5) | 156.5 (29.1-270.3) | -2.98 (-3.16--2.8) | -3.26 (-3.45--3.07) |
| Comoros | 7.3 (1.5-13.4) | 4.2 (0.8-7.8) | 213.6 (43.4-388.2) | 100.9 (20.5-184.8) | 21.8 (3.8-40.4) | 4.9 (0.8-9.1) | 598.3 (107.2-1097.5) | 114.6 (19.9-212.5) | 0.25 (0.12-0.38) | 0.13 (-0.02-0.27) |
| Congo | 46 (8.6-92.5) | 5.4 (1-10.7) | 1284.7 (254.1-2566.9) | 119.9 (22.5-241.8) | 114.7 (21.1-221.3) | 5.2 (0.9-10) | 3283.3 (661.8-6410.2) | 112.4 (20.6-214.9) | -0.98 (-1.36--0.59) | -1.15 (-1.56--0.73) |
| Cook Islands | 0.3 (0-0.5) | 2.4 (0.4-4.8) | 7.3 (1.4-14.4) | 55.7 (10.9-111.5) | 0 (0-0) | 0.1 (0-0.2) | 0.2 (0-0.8) | 1 (0.1-3.3) | -12.48 (-13.27--11.68) | -13.04 (-13.83--12.25) |
| Costa Rica | 233.3 (47.3-404.7) | 13.9 (2.8-24.4) | 5402.5 (1168.1-9028.7) | 299.4 (63.7-505.5) | 256.4 (47.4-448.3) | 4.5 (0.8-7.9) | 6001.4 (1204.8-10113) | 108.5 (21.9-182.8) | -3.76 (-3.94--3.58) | -3.54 (-3.75--3.34) |
| Coted'Ivoire | 299.7 (59.5-557.4) | 9.1 (1.7-17) | 8831.5 (1801.1-16066.2) | 203.6 (40.2-380.1) | 869.5 (188.6-1578.4) | 9.1 (1.9-16.6) | 24973.9 (5768.6-45456.6) | 202.1 (43.8-365.7) | 0.03 (-0.33-0.38) | -0.05 (-0.42-0.32) |
| Croatia | 1533.7 (280.5-2656.5) | 29.1 (5.3-50.8) | 31691.8 (6003.9-54058.4) | 552.7 (104.1-947.1) | 1001.7 (170.6-1808.2) | 10.4 (1.8-18.8) | 15972.7 (2839.5-28590.8) | 182.1 (33.9-321.7) | -3.62 (-3.75--3.49) | -3.91 (-4.05--3.77) |
| Cuba | 1625.4 (292.7-2822.6) | 16.9 (3-29.7) | 35563.5 (6650.6-60987.2) | 352.9 (65.7-607.6) | 1553.8 (281.2-2799.6) | 7.6 (1.4-13.6) | 31012.3 (5771.4-54270.1) | 161.7 (30.5-279.8) | -3.22 (-3.46--2.97) | -3.13 (-3.39--2.88) |
| Cyprus | 112 (20.5-198.4) | 20.6 (3.7-37.4) | 2256.2 (420.7-3910.9) | 340.8 (62-591.7) | 77.2 (13.4-146.1) | 4.7 (0.8-8.9) | 1447.5 (253.5-2682.4) | 79.4 (14.1-148.5) | -5.13 (-5.44--4.81) | -5 (-5.25--4.76) |
| Czechia | 3941.8 (758.8-6704.6) | 29.6 (5.7-50.3) | 85749.3 (16859.6-142375) | 641.5 (126.5-1063.7) | 2194.4 (380-3909) | 9.6 (1.7-16.9) | 36379.8 (6389.5-63826.1) | 172.3 (31.1-300) | -3.56 (-3.63--3.48) | -4.25 (-4.32--4.17) |
| Democratic People's Republic of Korea | 1323.6 (260.9-2391.2) | 9.9 (1.9-17.9) | 37292.6 (7944.1-65729.1) | 224.4 (45.8-397.9) | 4383.3 (818.2-7636.2) | 14.6 (2.6-25.2) | 113890.6 (23030.9-198968.6) | 349.3 (69.5-610) | 1.12 (0.79-1.46) | 1.25 (0.92-1.59) |
| Democratic Republic of the Congo | 1552.2 (300.1-2930) | 11.9 (2.2-22.4) | 44097.2 (8881.9-83306.3) | 271.9 (53-510.8) | 3650.8 (668.5-6725.1) | 11.7 (2.1-21.9) | 104348.8 (19306.2-190903.1) | 264.6 (48.5-489.6) | -0.32 (-0.51--0.14) | -0.35 (-0.54--0.16) |
| Denmark | 902.5 (161.1-1694.7) | 10.7 (1.9-19.9) | 16086.5 (2995.7-29453) | 203.6 (38.5-370.4) | 183.7 (33.1-355.1) | 1.4 (0.3-2.7) | 2970.1 (540.4-5620.4) | 25.9 (4.9-48.7) | -6.9 (-7.08--6.72) | -6.89 (-7.11--6.67) |
| Djibouti | 13 (2.9-22.8) | 10.5 (2.2-18.6) | 424.6 (95.8-743.1) | 258.3 (56.3-446.5) | 75.4 (17.3-138.2) | 13.1 (2.9-23.7) | 2377.7 (569.3-4331) | 314.4 (71.6-571.2) | 0.62 (0.45-0.8) | 0.54 (0.33-0.74) |
| Dominica | 5.3 (0.9-9.4) | 9.4 (1.7-16.7) | 106 (19.8-189.5) | 185 (35.1-330) | 3.1 (0.6-6.1) | 4.1 (0.7-8) | 65 (12.1-123) | 80.4 (15.1-153.4) | -2.91 (-3.22--2.6) | -2.88 (-3.21--2.54) |
| Dominican Republic | 558.1 (111.2-955.8) | 16.8 (3.3-29) | 15541.3 (3251.5-25739.2) | 387.8 (79.2-652.8) | 1098.5 (218.3-2016.6) | 11.1 (2.2-20.4) | 27991.8 (5917.5-51663.9) | 270.2 (57-496.6) | -0.77 (-0.98--0.56) | -0.67 (-0.83--0.51) |
| Ecuador | 537 (108-906.1) | 11.1 (2.2-19) | 13756 (2952.1-22382.6) | 242.1 (50.5-399.1) | 1143.2 (225.3-2036.5) | 7.6 (1.5-13.5) | 26255.3 (5447.7-45279.9) | 161 (33.1-279.1) | -1.44 (-1.96--0.92) | -1.59 (-2.07--1.11) |
| Egypt | 8319.2 (1762.5-13756.3) | 37 (7.5-62.5) | 251354.7 (55335.8-408468.5) | 838.5 (178.7-1384.9) | 9080.2 (1679.2-16133.9) | 18.1 (3.3-32.5) | 262320 (50704.4-459137.6) | 393.3 (73-693.3) | -2.07 (-2.2--1.95) | -2.35 (-2.46--2.25) |
| El Salvador | 480 (96.7-802.4) | 16.3 (3.2-27.4) | 12461.5 (2645.8-19975) | 394.2 (82.2-638.2) | 715.8 (135.2-1277.5) | 10.8 (2.1-19.3) | 15780.7 (3263.2-27554.7) | 251.8 (52.5-439.4) | -1.44 (-1.68--1.19) | -1.57 (-1.86--1.27) |
| Equatorial Guinea | 24.3 (4.7-43.8) | 14.2 (2.7-26.1) | 696.1 (139.5-1255.4) | 339.3 (66.5-609.4) | 4.3 (0.8-10.5) | 1.1 (0.2-2.6) | 113 (20.3-286) | 21.4 (3.9-51.9) | -12.29 (-14.29--10.24) | -13.1 (-15.18--10.96) |
| Eritrea | 125.1 (27.5-215.8) | 11.6 (2.4-20.6) | 4296.3 (961.5-7367.4) | 303.5 (66.2-523.1) | 286.4 (64.5-500.6) | 11.2 (2.4-19.6) | 9385 (2188.5-16498.5) | 276.2 (61.9-480.2) | -0.02 (-0.09-0.05) | -0.21 (-0.29--0.13) |
| Estonia | 430.5 (76.4-792.6) | 22.6 (4.1-41.5) | 7885.9 (1454.1-14369.9) | 399.8 (74.7-727.7) | 109.7 (17.8-213.8) | 3 (0.5-5.9) | 1267.9 (202.6-2469) | 36.5 (5.9-70.5) | -7.19 (-7.68--6.7) | -8.74 (-9.25--8.23) |
| Eswatini | 34.4 (7.2-58.7) | 13.6 (2.8-23.2) | 990.1 (216-1673.3) | 320.9 (67.8-546.7) | 77.2 (16.7-145) | 15 (3.1-28.2) | 2384.3 (533.7-4474.9) | 374.4 (80.6-697.9) | 0.84 (0.29-1.4) | 1.01 (0.36-1.67) |
| Ethiopia | 2650 (599.7-4479.7) | 13.8 (3-23.1) | 84158.8 (19897-140263.3) | 367.3 (83.7-619.8) | 3471.6 (748.6-5762.4) | 8.5 (1.8-14.4) | 100600.2 (22501.1-165689.2) | 203.6 (44.2-338) | -1.92 (-2.03--1.82) | -2.3 (-2.43--2.17) |
| Fiji | 29.7 (6-54.3) | 8.5 (1.6-15.8) | 1005.7 (213.2-1872.9) | 221.7 (44.9-406.2) | 32.2 (5.4-63.7) | 5.1 (0.9-10.1) | 944.8 (156.8-1896.2) | 118.9 (19.8-233.5) | -1.65 (-1.87--1.42) | -1.88 (-2.08--1.69) |
| Finland | 421.4 (68.3-852.8) | 5.9 (1-11.8) | 7722.5 (1334.2-15422.7) | 109.4 (19.2-218.4) | 184.6 (29.5-394.6) | 1.1 (0.2-2.3) | 2277.7 (356.8-4785.6) | 15.1 (2.4-31.2) | -5.7 (-5.86--5.53) | -6.66 (-6.92--6.39) |
| France | 2414.9 (430.6-4733.6) | 2.8 (0.5-5.4) | 40996.7 (7715.4-77642.1) | 50.1 (9.7-94.5) | 1193.1 (201.8-2453.7) | 0.6 (0.1-1.3) | 17186.2 (3034-35050.6) | 11.4 (2.1-23) | -4.96 (-5.07--4.85) | -4.85 (-4.96--4.73) |
| Gabon | 13.3 (2.2-29.9) | 2.8 (0.5-6.2) | 309.7 (53.5-684.8) | 56.6 (9.7-125.1) | 18.2 (3.4-39) | 2.3 (0.4-4.8) | 449.6 (87.4-958.6) | 44.2 (8.3-94.5) | -0.83 (-0.97--0.68) | -0.99 (-1.15--0.82) |
| Gambia | 19.3 (3.7-35.4) | 6.6 (1.2-11.7) | 522.2 (107.8-965.2) | 143.1 (27.9-260.7) | 66.5 (11.2-125.3) | 7.8 (1.3-14.6) | 1722.4 (297.3-3219.1) | 167.3 (28-314.8) | 0.41 (0.28-0.55) | 0.31 (0.14-0.48) |
| Georgia | 2517.6 (504.1-4287) | 43.8 (8.7-74.8) | 58073.8 (12016.1-97032.8) | 959.3 (198.6-1601.6) | 890.8 (163.5-1560.7) | 14.6 (2.7-25.3) | 19086.9 (3707.5-32774.7) | 343.7 (68.2-585) | -4.49 (-5.07--3.9) | -4.54 (-5.2--3.87) |
| Germany | 20371.9 (3822.5-36362.2) | 15.7 (3-27.8) | 386045.6 (75676-673341.3) | 312.9 (61.6-539.2) | 8760.7 (1684.4-16223.4) | 3.8 (0.7-7.1) | 138071 (26924.3-248668.7) | 71.7 (14.3-126.4) | -4.68 (-4.78--4.59) | -4.85 (-4.94--4.76) |
| Ghana | 380.4 (69-688.3) | 7.1 (1.2-13.2) | 10765.9 (2037.6-18954.2) | 161.4 (29.7-291) | 328.4 (59.1-656.5) | 2.4 (0.4-5) | 8816.9 (1691.7-17562.5) | 50.6 (9.3-100.8) | -4.01 (-4.37--3.66) | -4.24 (-4.6--3.87) |
| Greece | 1415.1 (246.5-2539) | 9.9 (1.7-17.7) | 29080.6 (5302.2-50638.4) | 203.7 (37.7-352.9) | 1277.3 (236.4-2326.2) | 4.6 (0.9-8.4) | 21936 (4319.2-39486.4) | 102.8 (20.7-181.6) | -2.73 (-2.92--2.54) | -2.43 (-2.56--2.3) |
| Greenland | 2.5 (0.5-4.6) | 9.1 (1.6-17.4) | 70.1 (14.6-127.1) | 193.3 (36.8-360.9) | 1.4 (0.2-2.7) | 2.5 (0.4-5) | 34.3 (6.4-67.5) | 51.9 (9.2-101.7) | -4.59 (-4.82--4.37) | -4.7 (-4.93--4.48) |
| Grenada | 6 (1.1-10.8) | 8.1 (1.5-14.3) | 134.3 (25.1-237) | 199.2 (37.7-350.2) | 2.7 (0.5-5.5) | 2.8 (0.5-5.6) | 62.6 (11.2-124.1) | 56.9 (10-111.8) | -3.75 (-4.05--3.45) | -4.17 (-4.52--3.82) |
| Guam | 0 (0-0.1) | 0.1 (0-0.3) | 0.8 (0-2.7) | 1.2 (0.1-4.3) | 0.1 (0-0.2) | 0 (0-0.1) | 1.5 (0.1-5.5) | 0.8 (0-2.8) | -2.96 (-3.31--2.61) | -1.7 (-2.03--1.37) |
| Guatemala | 590.8 (124.1-967) | 20.6 (4.2-34.5) | 17137.1 (3827.7-27466.2) | 456.9 (97-744.5) | 1139.5 (232-1952.9) | 11.9 (2.4-20.6) | 27575.8 (5751.6-45485.5) | 246.7 (50.8-413) | -1.8 (-2.25--1.35) | -2.12 (-2.65--1.59) |
| Guinea | 321.8 (65.6-566.7) | 10.6 (2.2-18.7) | 8068 (1669.1-14248.5) | 240.7 (49.8-424.9) | 579.6 (119.6-1020.9) | 11.4 (2.3-20.3) | 15166.2 (3284.7-26495.1) | 256.1 (53.3-449.8) | 0.56 (0.38-0.74) | 0.53 (0.35-0.72) |
| Guinea-Bissau | 81.5 (16.7-138.2) | 22.5 (4.5-38.7) | 2359.6 (503.7-4039) | 553.3 (114.8-940.4) | 146.3 (29.2-250.3) | 22.8 (4.3-39.3) | 4472.6 (933.9-7664.6) | 539.1 (106.4-925.2) | 0.14 (0.03-0.25) | 0.01 (-0.11-0.12) |
| Guyana | 46.6 (9-85.1) | 13.1 (2.4-24) | 1309.5 (268.6-2348.4) | 316.2 (62.4-569.3) | 26.5 (4.2-52.6) | 4.6 (0.8-9) | 692.4 (112.1-1404.6) | 105.1 (17-209.3) | -2.76 (-3.13--2.39) | -2.91 (-3.25--2.56) |
| Haiti | 1116.9 (226.7-1875.8) | 38.6 (7.7-65.1) | 32343.5 (6840.5-53412.2) | 935 (192.3-1564.4) | 1933.9 (444.4-3392.3) | 29.8 (6.5-53.2) | 56282 (13525.3-97216) | 705.5 (163.2-1232.8) | -0.69 (-0.77--0.62) | -0.75 (-0.84--0.66) |
| Honduras | 280.9 (56.9-480.8) | 15.3 (2.9-26.5) | 7739.3 (1669.7-13072.6) | 355.2 (74.4-600.9) | 1067.8 (214.6-1854.4) | 19.8 (3.9-34.4) | 25713.3 (5463.3-44151.7) | 408.2 (84.7-706.1) | 0.98 (0.76-1.19) | 0.59 (0.43-0.75) |
| Hungary | 3755.3 (714-6444.3) | 27.9 (5.3-47.8) | 87988 (17508.8-148045.7) | 639.5 (128.1-1072.4) | 2533 (456.2-4399.1) | 12.2 (2.2-21) | 45887.1 (8479.8-77989.8) | 244.5 (46.8-411.9) | -2.89 (-3.04--2.74) | -3.47 (-3.62--3.32) |
| Iceland | 2 (0.3-4.8) | 0.7 (0.1-1.6) | 29.6 (4.6-69.3) | 9.9 (1.6-23.3) | 0.9 (0.1-2.4) | 0.1 (0-0.3) | 10.8 (1.6-29.4) | 1.7 (0.3-4.3) | -5.73 (-6--5.45) | -6.11 (-6.4--5.81) |
| India | 92440.1 (20569.4-152257.6) | 20 (4.3-33.6) | 3017565.1 (697075.2-4871465.5) | 550.8 (124-901.5) | 176366.4 (34858-296207.8) | 15.4 (3-26.1) | 5066234.4 (1041340-8314562.1) | 398.3 (80.9-657.5) | -0.69 (-0.82--0.55) | -0.96 (-1.05--0.87) |
| Indonesia | 7949.5 (1613.6-13963.7) | 8.2 (1.6-14.9) | 261992.2 (56504.9-448169.1) | 222.5 (45.6-386.4) | 9277.7 (1835-17764.2) | 4.5 (0.8-8.4) | 278769.9 (58187.5-529778.2) | 107.1 (21.8-202.7) | -1.74 (-2--1.48) | -2.13 (-2.39--1.87) |
| Iran (Islamic Republic of) | 7211 (1500.6-11829.3) | 32.9 (6.7-55.8) | 205078 (44281.6-329001.5) | 748.1 (156.4-1217.5) | 10073.4 (1964.8-17597.9) | 14.2 (2.7-25.3) | 242572.8 (50015-411396.8) | 300 (59.9-514.6) | -3.12 (-3.34--2.9) | -3.34 (-3.55--3.13) |
| Iraq | 2820.8 (567.7-4794.9) | 36.1 (7.2-61.8) | 77123 (16062-127410.4) | 906.6 (185.5-1521) | 6163.2 (1220.9-10882.4) | 30.1 (5.8-52.5) | 165428.8 (35043.4-291814.3) | 654.3 (131.5-1150) | -1.27 (-1.48--1.07) | -1.68 (-1.88--1.49) |
| Ireland | 582.1 (110.1-1039.2) | 15 (2.8-26.5) | 11861.3 (2237.9-20827.3) | 302.6 (57-527.7) | 105.7 (20.2-206.2) | 1.3 (0.2-2.4) | 1667.6 (303.4-3167.8) | 21.2 (3.9-41.2) | -7.69 (-7.96--7.41) | -8.27 (-8.58--7.95) |
| Israel | 379.4 (66.6-683.8) | 8.4 (1.5-15.3) | 7502.2 (1332.7-13338.4) | 160.8 (29.2-285) | 143.4 (25.3-285.3) | 1 (0.2-2) | 2268.9 (426.3-4337.1) | 17.9 (3.4-34.8) | -7.1 (-7.3--6.89) | -7.33 (-7.57--7.09) |
| Italy | 5658.3 (1034.7-10129.9) | 6.6 (1.2-11.8) | 110701 (21155.5-191800.9) | 131.6 (25.7-228) | 3945.7 (766-7339.8) | 2.1 (0.4-3.8) | 57299.5 (11075.3-103228.5) | 38 (7.4-67.9) | -3.81 (-3.92--3.7) | -4.13 (-4.27--3.99) |
| Jamaica | 81 (14.9-144.9) | 4.5 (0.8-8) | 1652.8 (314-2855.1) | 93.9 (17.8-161.3) | 90.6 (16.2-169.7) | 2.7 (0.5-5) | 1870.2 (361.3-3415.4) | 59 (11.5-107.4) | -1.25 (-1.81--0.68) | -1.16 (-1.7--0.61) |
| Japan | 223.9 (40.9-543.2) | 0.2 (0-0.4) | 3875.6 (763.9-8898.7) | 2.5 (0.5-5.9) | 32.9 (3.1-104.5) | 0 (0-0) | 323.4 (35.9-1020.4) | 0.1 (0-0.2) | -10.92 (-12.14--9.68) | -12.19 (-13.75--10.59) |
| Jordan | 309.8 (67.7-524.4) | 25 (5.2-42.7) | 9407.5 (2116.9-15631.1) | 605.8 (132.6-1023.9) | 706.7 (140-1229.4) | 10.8 (2-19.2) | 20732.4 (4359.5-35325.9) | 246 (49.4-425.5) | -3.42 (-3.78--3.05) | -3.66 (-4.02--3.31) |
| Kazakhstan | 4262.5 (868-7052.8) | 37.2 (7.5-61.9) | 106428.2 (22448.3-172245) | 831.8 (173.4-1358.5) | 3405.7 (657.6-6040.4) | 23.9 (4.5-42.7) | 73892.8 (14883.7-128623.1) | 442.7 (87.3-779.8) | -2.79 (-3.51--2.07) | -3.55 (-4.38--2.71) |
| Kenya | 305.5 (65.8-538.7) | 4.2 (0.9-7.5) | 8547.5 (1911.5-14778.5) | 98 (21.3-171.5) | 1190.9 (246.2-2082.3) | 6.1 (1.2-10.8) | 34414.9 (7505-59454.3) | 138.1 (28.8-241.2) | 1.56 (1.17-1.95) | 1.45 (0.99-1.92) |
| Kiribati | 0.4 (0.1-0.8) | 1.1 (0.2-2.5) | 11.8 (1.9-26.1) | 28.3 (4.5-61.1) | 0.6 (0.1-1.6) | 1.1 (0.2-2.6) | 20.7 (4.3-49.1) | 25.7 (5.1-60.6) | 0.01 (-0.18-0.2) | -0.11 (-0.33-0.1) |
| Kuwait | 116.4 (23.5-198.7) | 18.9 (3.7-33.3) | 3884.3 (820.2-6379.5) | 459.8 (91.9-799.2) | 228.2 (43.1-409.2) | 7.3 (1.2-13.5) | 7415.9 (1503.7-13319.8) | 175.2 (32.4-311.2) | -3.43 (-3.86--3) | -3.47 (-3.89--3.04) |
| Kyrgyzstan | 1041 (220.6-1720.5) | 38.5 (8-64.4) | 25746.4 (5741.4-42508.1) | 866.1 (191.1-1426.2) | 1486.6 (317.2-2487.8) | 37.6 (7.9-64.1) | 34668.5 (7713.6-57308.7) | 738.5 (160.4-1218.4) | -0.01 (-0.42-0.41) | -0.73 (-1.19--0.27) |
| Lao People's Democratic Republic | 380.2 (76.9-677.9) | 19.7 (3.9-35.1) | 12002.1 (2571-21093.7) | 518.1 (106.8-916.9) | 127.5 (23-257.2) | 3.3 (0.6-6.7) | 3615.4 (662.4-7276.6) | 71.5 (13-144.4) | -6.12 (-6.34--5.9) | -6.75 (-7--6.49) |
| Latvia | 676.8 (118.4-1218.2) | 19.8 (3.5-35.9) | 13835.8 (2504.8-24130) | 401.5 (72.6-697.2) | 279.4 (47.8-541.5) | 6.1 (1-11.7) | 4516.5 (770.6-8571.2) | 114.1 (19.7-218) | -4.65 (-5.21--4.08) | -5.22 (-5.87--4.57) |
| Lebanon | 576.4 (117.1-1012.6) | 28.9 (5.7-51.5) | 15698.7 (3285.5-26827.2) | 694.8 (144-1199.6) | 575.9 (117.8-1023.1) | 8.9 (1.8-15.8) | 11783.9 (2514.4-20276.1) | 191.4 (40.8-328.8) | -4.11 (-4.46--3.75) | -4.42 (-4.85--3.99) |
| Lesotho | 60.3 (13.1-100.1) | 8 (1.7-13.3) | 1509.9 (338.1-2510.8) | 178.9 (39.5-298) | 140.6 (31.3-265.8) | 14.5 (3.1-26.7) | 4044.2 (931.5-7677.4) | 354.5 (79.7-670.3) | 2.87 (2.3-3.44) | 3.13 (2.52-3.74) |
| Liberia | 109.8 (21.6-187.6) | 10.8 (2.1-18.7) | 2785.9 (572.1-4784.8) | 240.2 (48.7-412.5) | 264.9 (58.9-480) | 14.2 (3-25.8) | 7579 (1794.4-13860.8) | 317.1 (70.3-575.7) | 0.5 (0.2-0.81) | 0.51 (0.2-0.82) |
| Libya | 282.1 (56.2-493.3) | 15.3 (3-27.1) | 7902 (1665.1-13571.8) | 371.4 (75.5-640.1) | 952.4 (221.8-1684.3) | 18.7 (4-33.8) | 28096.4 (6881.1-49087) | 452.7 (106.8-795.2) | 0.92 (0.7-1.14) | 0.79 (0.56-1.03) |
| Lithuania | 582.5 (102.7-1102.7) | 13.4 (2.3-25.3) | 10875.5 (2138.7-20359.4) | 248.5 (48.9-460.4) | 180.9 (32-383.1) | 2.6 (0.5-5.5) | 2473.3 (431.9-5318.4) | 39.6 (7.1-87.2) | -6.03 (-6.63--5.42) | -6.79 (-7.46--6.12) |
| Luxembourg | 40.7 (7.3-74.7) | 7.8 (1.4-14.2) | 790 (153.1-1422.2) | 150.6 (29.5-269.8) | 15.5 (2.8-29.9) | 1.3 (0.2-2.4) | 244.3 (44.6-468.3) | 22 (4-41.7) | -5.94 (-6.16--5.72) | -6.41 (-6.66--6.16) |
| Madagascar | 468.4 (100.8-790.1) | 9.7 (2.1-16.6) | 14384.4 (3255.2-23851.2) | 252.2 (54.9-424.8) | 1298.1 (275.1-2295.8) | 12.2 (2.4-22.2) | 42811.1 (9363.2-74282.8) | 307.8 (64.2-545.9) | 0.35 (0.16-0.54) | 0.27 (0.08-0.46) |
| Malawi | 231.4 (46.3-402) | 6.7 (1.3-11.7) | 7017 (1451-12106.1) | 165.1 (33.6-287.8) | 577.4 (111.6-988.3) | 8.3 (1.6-14.4) | 17912.8 (3654.2-30934.2) | 206.4 (39.9-353.4) | 0.36 (0.01-0.71) | 0.35 (-0.04-0.73) |
| Malaysia | 283.3 (53.8-562) | 3.3 (0.6-6.5) | 7276.3 (1455.6-14145.3) | 73.4 (14.2-145.3) | 97.1 (16.3-241) | 0.4 (0.1-1) | 2253.1 (386.6-5707.2) | 8.2 (1.4-20.2) | -6.24 (-6.48--6) | -6.57 (-6.77--6.38) |
| Maldives | 0 (0-0.1) | 0.1 (0-0.2) | 1.2 (0.1-3.9) | 1.2 (0.1-3.8) | 0 (0-0) | 0 (0-0) | 0 (0-0.2) | 0 (0-0) | -15.15 (-15.46--14.84) | -16.82 (-17.28--16.36) |
| Mali | 212.2 (41.5-373.1) | 6.3 (1.2-11.3) | 5871.2 (1159.9-10329.6) | 143.1 (28.2-251.7) | 363.2 (66.6-664.3) | 4.8 (0.9-8.9) | 10041 (1914.1-18302) | 107.4 (19.9-196.9) | -0.79 (-0.88--0.69) | -0.87 (-0.97--0.77) |
| Malta | 53.5 (9.3-93.7) | 13.4 (2.3-23.3) | 1102.3 (201.9-1921.7) | 263.5 (48-459.2) | 20.6 (3.5-41.3) | 1.9 (0.3-3.7) | 320.7 (54.1-631.6) | 33.7 (5.7-66) | -6.12 (-6.31--5.92) | -6.44 (-6.59--6.28) |
| Marshall Islands | 1.3 (0.3-2.4) | 8.7 (1.9-16.6) | 41.7 (9.7-76.7) | 219.8 (49.9-413.8) | 2.5 (0.5-4.9) | 7.7 (1.6-15.1) | 87.4 (18.3-168.6) | 203 (42.4-390.2) | -0.11 (-0.3-0.07) | 0.04 (-0.17-0.25) |
| Mauritania | 124.2 (23.2-221.9) | 13.8 (2.6-24.9) | 3156.6 (605.9-5553.6) | 312.1 (59.1-551.9) | 179.2 (37.4-327.4) | 9.4 (1.9-17.5) | 4366.5 (960.1-7870) | 199.7 (42.6-360.4) | -1.64 (-1.83--1.45) | -1.84 (-2.03--1.66) |
| Mauritius | 130.5 (24.3-225.6) | 19.5 (3.6-34.4) | 3744 (724.7-6361.4) | 475.3 (90-817.4) | 78.2 (14.4-142.4) | 4.7 (0.9-8.5) | 1904.2 (366.8-3395.9) | 112.1 (21.8-198.3) | -4.88 (-5.11--4.64) | -4.82 (-5.05--4.59) |
| Mexico | 3610 (714.6-6183.7) | 10 (1.9-17.4) | 89945.8 (18334.7-150669.2) | 205.8 (41.1-350) | 10164.2 (1960.1-18352.5) | 8.7 (1.6-15.8) | 235150.3 (48384.7-408802.3) | 184.7 (37.6-323.3) | -0.62 (-0.91--0.33) | -0.56 (-0.85--0.27) |
| Micronesia (Federated States of) | 4.7 (0.9-8.7) | 10.3 (1.9-19.2) | 144.7 (29.3-269.3) | 268.8 (52.3-503.9) | 5.6 (1.1-11.1) | 8.6 (1.6-16.7) | 180.9 (37.2-341.8) | 219.6 (43.5-424.7) | -0.52 (-0.61--0.44) | -0.57 (-0.66--0.48) |
| Monaco | 1.6 (0.3-3.3) | 2.1 (0.4-4.1) | 26.7 (4.8-52.7) | 39.6 (7.5-76.2) | 0.3 (0-0.6) | 0.2 (0-0.5) | 3.7 (0.5-8.8) | 3.7 (0.6-8.7) | -7.43 (-7.83--7.03) | -7.83 (-8.25--7.41) |
| Mongolia | 452.1 (94.4-768.2) | 49.7 (10.2-84.8) | 11090.6 (2432.3-18989.4) | 1060.8 (225.9-1810) | 561.3 (118.3-928.8) | 30.7 (6.2-52.2) | 14819.3 (3268.8-24283.1) | 625.2 (132.8-1033.7) | -2.05 (-2.36--1.74) | -2.18 (-2.53--1.83) |
| Montenegro | 100.4 (20.1-175.1) | 16.9 (3.4-29.8) | 2402.3 (494.9-4039.1) | 382.8 (78.4-646.2) | 146.5 (26.5-263.5) | 17.3 (3.2-31.1) | 2851.3 (545.2-4988.4) | 315.6 (60.7-550.9) | -0.17 (-0.43-0.1) | -0.95 (-1.27--0.63) |
| Morocco | 4879.7 (1010.7-8500.2) | 36.1 (7.3-63.4) | 134219 (29015.9-228184.4) | 882.7 (188.2-1513.5) | 7585.6 (1557-13854.3) | 24.6 (4.9-44.8) | 188783 (40790.3-337102.2) | 540.8 (114.7-979.4) | -1.34 (-1.39--1.3) | -1.73 (-1.8--1.67) |
| Mozambique | 259.5 (53.9-435) | 5.1 (1-8.6) | 7512.1 (1635.9-12309) | 120.4 (25.6-200.6) | 663.1 (134.2-1137.9) | 6.6 (1.3-11.5) | 20634.2 (4388.7-35229.4) | 162.6 (33.3-278.7) | 1.27 (1.08-1.45) | 1.43 (1.21-1.64) |
| Myanmar | 4006.8 (834.8-7148.5) | 18.7 (3.8-33.4) | 123883 (27432.7-217818.2) | 482.3 (103-857.5) | 759.1 (123.9-1601) | 1.9 (0.3-3.9) | 18264.9 (2970.5-37972.1) | 38.5 (6.3-80) | -8.23 (-8.78--7.66) | -9.03 (-9.65--8.41) |
| Namibia | 63.7 (13.7-114.4) | 11.6 (2.5-21.1) | 1774.4 (390-3135.1) | 268.4 (57.9-480.2) | 109.6 (22.7-195.3) | 9.2 (1.9-16.2) | 3002.6 (642.9-5369.8) | 206.4 (43.3-367.7) | -1.12 (-1.47--0.77) | -1.26 (-1.66--0.87) |
| Nauru | 0 (0-0.1) | 0.5 (0.1-1.4) | 0.8 (0.1-2.4) | 14.3 (1.9-38.2) | 0.2 (0-0.3) | 2.9 (0.5-6.6) | 5.4 (1.1-11.8) | 75.1 (14.5-166.5) | 4.71 (1.92-7.58) | 4.64 (1.76-7.6) |
| Nepal | 1956.7 (465.8-3208.6) | 21.4 (4.9-35.7) | 62047.6 (15281.1-100878.7) | 571.5 (137.4-934) | 4060.4 (831.5-7074.7) | 18.9 (3.7-33.4) | 111936.8 (24360.2-188096.9) | 459.9 (97.7-784.6) | -0.11 (-0.29-0.06) | -0.44 (-0.61--0.26) |
| Netherlands | 1721.6 (308.4-3138.9) | 8.6 (1.5-15.5) | 34169 (6247.5-61880.6) | 175.8 (32.3-316.6) | 580.1 (95.8-1134.1) | 1.5 (0.2-2.9) | 8818.5 (1540.6-16770.3) | 24.7 (4.4-47.1) | -6.18 (-6.45--5.9) | -6.8 (-7.06--6.54) |
| New Zealand | 438.4 (78.9-796.3) | 11.5 (2.1-20.6) | 9225.8 (1719.8-16343.3) | 241.9 (45.5-424.8) | 214.6 (38.6-402.5) | 2.4 (0.4-4.4) | 3574.9 (690.1-6446.4) | 43.6 (8.7-77.1) | -5.35 (-5.45--5.24) | -5.83 (-6.03--5.64) |
| Nicaragua | 177.3 (38.2-295.5) | 12.5 (2.6-21.4) | 4680.2 (1069.2-7624.2) | 281.8 (62.1-462.5) | 453.7 (98.5-769.7) | 10.4 (2.2-17.7) | 11127.1 (2550.3-18336.5) | 221.2 (49.8-366.7) | -0.4 (-0.61--0.2) | -0.67 (-0.84--0.51) |
| Niger | 226.6 (45.4-409.4) | 9.8 (1.9-17.8) | 6331.7 (1325.4-11229.9) | 220.2 (44-396.1) | 665.6 (150.5-1186.5) | 9.7 (2.1-17.4) | 17829.5 (4198.7-31196.3) | 212.5 (48.5-376) | 0.01 (-0.09-0.11) | -0.09 (-0.2-0.02) |
| Nigeria | 4448.8 (892.8-8006.1) | 11.6 (2.3-21.1) | 112606.1 (23297.6-199908.1) | 251.5 (51.1-452) | 6329.9 (1294.1-11105.5) | 8.3 (1.6-14.8) | 164032.6 (35646.3-284584.3) | 172.4 (35.7-302.9) | -1.51 (-1.76--1.25) | -1.7 (-1.98--1.43) |
| Niue | 0.1 (0-0.1) | 2.5 (0.4-4.9) | 1.2 (0.2-2.6) | 55 (9.6-118.8) | 0 (0-0) | 0.9 (0.2-2.3) | 0.4 (0.1-0.9) | 19.5 (3.8-45.3) | -4.3 (-4.81--3.79) | -4.57 (-5.12--4.03) |
| North Macedonia | 445.6 (96.1-737.7) | 25.6 (5.4-42.7) | 11181.8 (2476.8-17925.1) | 588.8 (129.5-952.1) | 506.1 (91.5-886.5) | 20.5 (3.7-36.4) | 10761.4 (2030.8-18680.4) | 368.3 (69.5-639.2) | -1.17 (-1.65--0.68) | -1.96 (-2.35--1.57) |
| Northern Mariana Islands | 0 (0-0) | 0.1 (0-0.2) | 0.3 (0-1.1) | 1.3 (0.1-4.3) | 0.1 (0-0.2) | 0.2 (0-0.6) | 2.1 (0.3-6) | 4.4 (0.7-12.6) | 5.87 (4.63-7.13) | 6.29 (5.02-7.57) |
| Norway | 353.6 (61.1-686.2) | 4.8 (0.8-9.3) | 6266 (1144.2-12005.5) | 94.8 (18.1-179.9) | 67.9 (12-144.5) | 0.6 (0.1-1.2) | 969.3 (182.4-2032.7) | 9.1 (1.8-19) | -7.3 (-7.46--7.13) | -7.81 (-8.03--7.59) |
| Oman | 113.1 (21.6-203.9) | 18 (3.3-33) | 3257.9 (653.3-5959.7) | 417.9 (79.7-755.5) | 131.8 (26.1-245.8) | 8.1 (1.5-15.1) | 3894.1 (814-7183.5) | 163.5 (31.4-305.5) | -2.72 (-2.89--2.54) | -3.22 (-3.35--3.09) |
| Pakistan | 11543 (2494.8-20346) | 21.3 (4.5-37.5) | 338413.1 (76131.4-579184) | 552.3 (121.2-963.9) | 29989.8 (6481.2-52583.6) | 25.2 (5.3-44.6) | 942733.1 (210904.6-1619260.2) | 646.2 (140.6-1129.1) | 0.48 (0.34-0.63) | 0.4 (0.23-0.57) |
| Palau | 0.1 (0-0.2) | 1.2 (0.2-2.9) | 2.9 (0.5-6.4) | 27.5 (4.9-61.3) | 0.1 (0-0.2) | 0.6 (0.1-1.6) | 2.6 (0.4-6.5) | 12.8 (2-32.8) | -1.84 (-2.09--1.6) | -2.18 (-2.44--1.92) |
| Palestine | 305.2 (58.1-533) | 39.7 (7.4-69.3) | 7634.2 (1579.7-13062.2) | 865.3 (172.9-1490.8) | 499 (111-813.2) | 23.9 (5.1-39.7) | 13297.8 (3117.2-21269.4) | 496.8 (111.4-806.2) | -1.74 (-2--1.49) | -1.91 (-2.12--1.69) |
| Panama | 138.2 (25.9-238.9) | 9.9 (1.8-17.2) | 3145.5 (626.3-5239.5) | 207.7 (40.5-349.7) | 155.1 (28.4-278.8) | 3.4 (0.6-6.1) | 3419 (652.9-5936.3) | 76.6 (14.6-133) | -3.37 (-3.59--3.16) | -3.19 (-3.43--2.96) |
| Papua New Guinea | 183.3 (37.8-321.3) | 10.3 (2.1-18.3) | 6293.9 (1321.2-11254.1) | 283.1 (57.9-498.4) | 463.1 (81.4-823.9) | 9.1 (1.5-16.5) | 15834.3 (2929.9-28475.1) | 242.9 (42.6-431.2) | -0.18 (-0.47-0.1) | -0.28 (-0.56-0) |
| Paraguay | 252.3 (47.8-433.5) | 11.9 (2.2-20.7) | 6368.5 (1297.1-10701.7) | 273.9 (54.4-463.1) | 429.2 (80.6-780.4) | 7.6 (1.4-13.9) | 10603.1 (2093.4-19016.6) | 176.2 (34.3-316.6) | -1.13 (-1.32--0.94) | -1.22 (-1.37--1.07) |
| Peru | 538 (102.8-939.8) | 4.7 (0.9-8.3) | 13700.1 (2686-23554) | 105.7 (20.3-183.3) | 542.4 (95.3-1083.4) | 1.6 (0.3-3.2) | 12315.6 (2366.9-23940.4) | 35.2 (6.7-69.1) | -4.36 (-4.75--3.96) | -4.35 (-4.73--3.97) |
| Philippines | 1246.7 (228.9-2283.5) | 5.1 (0.9-9.8) | 38841.7 (7701.3-70424) | 113.9 (21.2-208.9) | 1617.4 (299.4-3374.8) | 2.2 (0.4-4.7) | 46591.4 (8838.3-96137.9) | 52.3 (9.9-108.1) | -2.55 (-2.82--2.28) | -2.47 (-2.74--2.19) |
| Poland | 11227.9 (2190.1-19310.9) | 27.4 (5.4-47.1) | 268539.9 (54480.4-449421) | 632.6 (128.5-1059.6) | 5840.6 (1077.3-10784.6) | 7.6 (1.4-14) | 102093.1 (19487.6-183851.5) | 144.6 (28.1-256.8) | -4.47 (-4.6--4.34) | -5.08 (-5.23--4.94) |
| Portugal | 375.6 (63.3-743.7) | 3.2 (0.5-6.2) | 7023.9 (1213.8-13444.7) | 55.9 (9.7-106.9) | 162.8 (29.2-341) | 0.5 (0.1-1.1) | 2493.7 (446.6-5241.2) | 10.3 (1.9-21.9) | -5.9 (-6.23--5.58) | -5.66 (-6.1--5.22) |
| Puerto Rico | 240.6 (44.4-446.5) | 7.2 (1.3-13.4) | 5095.9 (963.3-9195.6) | 145.1 (27.5-261.7) | 102.9 (16.7-210.5) | 1.2 (0.2-2.4) | 1818.4 (298.4-3553.6) | 27.8 (4.7-54.2) | -5.99 (-6.43--5.55) | -5.74 (-6.22--5.27) |
| Qatar | 16.2 (3.2-30.1) | 19.8 (3.6-35.9) | 538.1 (108.2-968.9) | 383.9 (71.4-723.2) | 19.7 (3.9-39.8) | 3.2 (0.5-6.5) | 696.4 (138.7-1375.1) | 56.5 (9.6-117.1) | -7.55 (-8.32--6.78) | -7.81 (-8.5--7.11) |
| Republic of Korea | 276.2 (49.4-565.4) | 1.4 (0.3-2.9) | 6883.2 (1296.3-13689.7) | 24.8 (4.6-50.6) | 107.4 (16.8-272.3) | 0.1 (0-0.3) | 1624.6 (265.4-3911.7) | 1.9 (0.3-4.5) | -8.03 (-8.23--7.82) | -8.41 (-8.57--8.25) |
| Republic of Moldova | 1385.5 (270.1-2384.2) | 39.8 (7.5-69.3) | 30042.7 (6037.9-50622.7) | 743.3 (148.6-1260.5) | 1307.6 (244.4-2273.7) | 21.8 (4.1-37.9) | 26620 (5340.3-45272.1) | 457.7 (92.4-775.8) | -2.77 (-3.27--2.26) | -2.3 (-2.85--1.76) |
| Romania | 6986.6 (1370.7-11908.5) | 30 (5.8-51.3) | 156207.5 (32338.9-259935.8) | 602.3 (123.5-1007.1) | 5364.4 (1009.7-9777.4) | 13.7 (2.6-24.9) | 99457.3 (18689.6-179185.4) | 282.5 (53.7-502.6) | -3.27 (-3.58--2.97) | -3.34 (-3.7--2.98) |
| Russian Federation | 28517.5 (5376.1-50113.4) | 18.3 (3.4-32.7) | 637859.3 (127243.7-1104676.5) | 376.6 (74.7-657.5) | 21865.9 (4247.1-40923.7) | 9.2 (1.8-17.2) | 427147.9 (84994.2-794607.4) | 186.3 (37.5-347) | -3.29 (-4.14--2.44) | -3.58 (-4.53--2.61) |
| Rwanda | 412.9 (90.9-732.1) | 15.8 (3.5-28.8) | 12589.3 (2819.5-22125.6) | 401.4 (88.4-709.8) | 503.1 (108.4-919.4) | 9.2 (1.9-16.8) | 14331.8 (3177.5-25680.5) | 209.6 (45.6-379.5) | -2.79 (-3.18--2.4) | -3.27 (-3.69--2.83) |
| Saint Kitts and Nevis | 4.2 (0.8-7.7) | 12.1 (2.3-22) | 90.5 (17.1-159.9) | 261.1 (50.9-456.6) | 1.4 (0.3-2.7) | 2.5 (0.4-4.9) | 30.7 (6-59.9) | 47.5 (9.1-92.9) | -4.96 (-5.27--4.65) | -5.57 (-5.94--5.2) |
| Saint Lucia | 6.6 (1.1-12) | 9.1 (1.6-16.6) | 143.4 (25.9-251.4) | 175.3 (31.5-311.4) | 4.1 (0.7-7.8) | 1.8 (0.3-3.4) | 85.4 (15.6-159.6) | 36.2 (6.6-67.7) | -5.82 (-6.3--5.35) | -5.48 (-5.92--5.04) |
| Saint Vincent and the Grenadines | 11 (2.1-19.3) | 16.8 (3.2-29.5) | 252.8 (51-431.8) | 363.2 (73-620.5) | 8.3 (1.5-15.3) | 6.6 (1.2-12) | 180.2 (34-325) | 132.9 (24.9-238.6) | -3.29 (-3.53--3.05) | -3.53 (-3.74--3.31) |
| Samoa | 3.9 (0.8-7.4) | 5.3 (1-10.2) | 105.9 (20.4-209.8) | 121.6 (23.5-235.7) | 3.1 (0.6-6.5) | 2.5 (0.5-5.3) | 83.1 (16.9-169.9) | 56.1 (11.5-114) | -2.97 (-3.33--2.61) | -3.04 (-3.44--2.63) |
| San Marino | 1.4 (0.2-2.7) | 3.9 (0.7-7.4) | 25 (4.4-46.8) | 72.1 (12.7-132.1) | 1 (0.2-2) | 1 (0.2-2) | 15.7 (3.1-30.4) | 20 (4-38.9) | -4 (-4.72--3.27) | -3.83 (-4.53--3.13) |
| Sao Tome and Principe | 2.7 (0.5-4.9) | 4.8 (0.9-8.6) | 63.9 (11.7-111.9) | 101.2 (18.6-178.7) | 4.3 (0.8-8.3) | 4.5 (0.9-8.9) | 110.3 (21.6-209.3) | 94.3 (18.6-181.5) | -0.32 (-0.61--0.03) | -0.51 (-0.88--0.14) |
| Saudi Arabia | 1376.8 (277.7-2382.6) | 24.1 (4.7-41.9) | 42290.1 (8790.9-72771.2) | 593.5 (119.4-1026.7) | 3468.5 (681.7-6093.1) | 15.7 (2.9-28.4) | 128394.5 (25901.1-226670.1) | 409.3 (78.5-735.9) | -1.6 (-1.84--1.36) | -1.28 (-1.52--1.03) |
| Senegal | 153.5 (27.9-287.6) | 5.5 (1-10.3) | 3940.8 (728.9-7367.4) | 120 (21.9-224.2) | 303.4 (59.5-580.3) | 4.6 (0.9-8.7) | 7470.6 (1481.6-14011.6) | 95.4 (19-181.7) | -0.76 (-0.92--0.6) | -0.88 (-1.05--0.72) |
| Serbia | 2947.1 (589.2-4972.1) | 34 (6.7-58) | 65516.8 (13712.6-108762.8) | 646.8 (133.5-1071.4) | 2889.6 (576.7-5090.7) | 17.1 (3.5-30) | 51300.5 (10434.7-89768.8) | 321.2 (66.6-559.6) | -3.11 (-3.48--2.73) | -3.05 (-3.41--2.69) |
| Seychelles | 1.1 (0.2-2.2) | 2 (0.4-4) | 25.9 (5.1-51.1) | 45.5 (9.1-90.5) | 0.4 (0.1-1) | 0.4 (0.1-1) | 9.6 (1.6-22.1) | 8.6 (1.4-19.6) | -4.19 (-4.58--3.79) | -4.61 (-4.96--4.25) |
| Sierra Leone | 146.4 (27.3-268.9) | 8 (1.5-14.7) | 3619.8 (709-6561.8) | 174.7 (33.6-315.9) | 230.3 (41.7-433.5) | 6.9 (1.2-13.1) | 6135.8 (1135.2-11389.8) | 151.8 (27.6-285.3) | -0.44 (-0.89-0.01) | -0.42 (-0.88-0.05) |
| Singapore | 48.6 (8.7-99.1) | 2.4 (0.4-5) | 1276.8 (241.5-2548.8) | 53.6 (9.9-107.9) | 3.4 (0.4-9.9) | 0 (0-0.1) | 73.8 (9.2-211.5) | 0.9 (0.1-2.5) | -13.25 (-13.66--12.85) | -13.26 (-13.61--12.92) |
| Slovakia | 2091.8 (405-3602.5) | 36.6 (7.1-63.1) | 46560.3 (9398.6-78439.5) | 797.9 (161.8-1339.2) | 1508.6 (281.9-2699.4) | 16.1 (3-28.8) | 26945.1 (5275.9-46967) | 291.8 (58.2-504.5) | -2.74 (-2.87--2.62) | -3.4 (-3.56--3.25) |
| Slovenia | 326.9 (63.1-571.6) | 13.5 (2.6-23.7) | 7049.3 (1391.9-12038.3) | 289.4 (57.3-494.2) | 199.8 (34.2-354) | 3.9 (0.7-6.7) | 3386.8 (617-5888.6) | 75.9 (14.4-131.1) | -4.72 (-4.96--4.47) | -4.99 (-5.23--4.76) |
| Solomon Islands | 11.8 (2.1-22.8) | 9.8 (1.7-19.2) | 387.2 (71.2-744.9) | 247.3 (43.8-474.6) | 32.8 (6.9-62.8) | 10 (2-19.2) | 1076.3 (222.3-2069.7) | 254.7 (53.4-489.3) | 0.55 (0.26-0.84) | 0.61 (0.31-0.91) |
| Somalia | 244.9 (52.1-448.2) | 10.6 (2.1-18.7) | 8224.7 (1785.3-15144.6) | 274.7 (57.8-505.8) | 644.7 (140.8-1178.4) | 11 (2.3-20.6) | 21576.6 (4818.6-39438.7) | 282.4 (60.6-513) | 0.21 (0.06-0.35) | 0.1 (-0.04-0.24) |
| South Africa | 1674.1 (366.4-2889.3) | 8.3 (1.8-14.6) | 51171.9 (11746.6-84209.3) | 217.6 (48.9-366) | 3307.8 (673.7-5719.8) | 8 (1.6-13.9) | 89490.4 (19356.4-151382.6) | 184.1 (38.8-313.5) | -0.38 (-0.81-0.05) | -0.75 (-1.18--0.33) |
| South Sudan | 193.3 (36.9-350.4) | 8.1 (1.6-14.6) | 5434.6 (1064.3-9837.4) | 201 (38.6-361.2) | 394.7 (83.4-728.8) | 10.9 (2.2-20.2) | 12164.4 (2687.5-22142.2) | 269.8 (57.1-496.8) | 0.68 (0.46-0.89) | 0.67 (0.42-0.92) |
| Spain | 1553.1 (284.7-3073.9) | 3 (0.6-5.9) | 28345.1 (5135.3-53767.5) | 55.6 (10.3-104.2) | 709.4 (125.8-1525.8) | 0.6 (0.1-1.2) | 10650.3 (1954.8-22312.7) | 10.5 (2-21.5) | -5.53 (-5.76--5.3) | -5.54 (-5.83--5.25) |
| Sri Lanka | 1053.6 (210.1-1825.2) | 10.9 (2.1-19.1) | 29798.1 (6189.8-50904.1) | 255.5 (51.8-438.9) | 746.6 (126.3-1479.2) | 3.1 (0.5-6.1) | 17681.1 (3054-33922.3) | 68.3 (11.8-130.7) | -3.8 (-4.14--3.45) | -4.14 (-4.51--3.76) |
| Sudan | 4632.6 (988.4-7766.9) | 52.5 (11-88.7) | 137534.2 (30615.1-231469.8) | 1346.9 (291.8-2265.4) | 6480.8 (1396.6-11637.3) | 34.8 (7.2-63.4) | 191028.4 (42649.9-337333.7) | 835.9 (181.2-1491.9) | -1.59 (-1.68--1.5) | -1.8 (-1.89--1.71) |
| Suriname | 42.4 (8-72.2) | 17.1 (3.2-29.7) | 1127.4 (224.5-1884.4) | 418.2 (81.7-706.5) | 45.8 (9.7-84.5) | 7.4 (1.6-13.6) | 1207.9 (272.3-2202.7) | 186 (41.7-338) | -2.87 (-3.15--2.59) | -2.88 (-3.18--2.59) |
| Sweden | 1247.1 (213-2319.6) | 7.6 (1.3-14) | 20540.3 (3520.8-38038.3) | 136.1 (23.7-249.4) | 361.4 (61-765.1) | 1.3 (0.2-2.7) | 4831.3 (844.1-10020) | 20 (3.7-41.7) | -5.82 (-5.91--5.73) | -6.24 (-6.34--6.14) |
| Switzerland | 1109.6 (209.8-2027.5) | 10.1 (1.9-18.3) | 20165.5 (3831.2-35701.1) | 197.3 (38-346.6) | 467 (83.7-879.5) | 2 (0.4-3.7) | 6618.1 (1264-12214.8) | 33.6 (6.7-60.8) | -5.48 (-5.6--5.36) | -5.92 (-6.02--5.83) |
| Syrian Arab Republic | 2675.1 (639.9-4444) | 53.4 (12.4-89.7) | 78744.7 (19555.4-128830.4) | 1319 (321.5-2175.4) | 5424.5 (1219.4-9326.1) | 49 (10.5-84.5) | 143430 (34288.3-246559) | 1072.4 (248.5-1832.7) | -0.65 (-0.79--0.5) | -1.05 (-1.24--0.86) |
| Taiwan (Province of China) | 235 (39.1-454.9) | 2.1 (0.3-4) | 5481.8 (955.7-10421.7) | 37.9 (6.4-73.1) | 125.2 (21.6-272.1) | 0.3 (0.1-0.6) | 2356.7 (440.5-4976.8) | 5.9 (1.1-12.4) | -5.88 (-6.17--5.58) | -5.4 (-5.69--5.1) |
| Tajikistan | 1180.6 (242.3-1978.9) | 46.5 (9.4-78.5) | 29139.6 (6308.4-48342.6) | 1043.1 (221.5-1724.3) | 1652.5 (323.5-2785.3) | 36 (6.9-61.7) | 42346.4 (8525.8-70517.3) | 730.3 (144.1-1228) | -1.18 (-1.55--0.81) | -1.58 (-1.91--1.25) |
| Thailand | 1202.2 (231.9-2210.1) | 4 (0.8-7.3) | 32617.3 (6506.6-58410.5) | 86.3 (16.9-156.9) | 703.1 (122.1-1516.6) | 0.7 (0.1-1.4) | 15289.3 (2799-32290.9) | 15.3 (3-32.4) | -6.04 (-6.37--5.72) | -5.9 (-6.25--5.55) |
| Timor-Leste | 45.5 (10.9-75.1) | 17.3 (3.9-29.2) | 1487.1 (370.2-2426.5) | 422.7 (100.4-699.6) | 149.5 (30.3-264.1) | 19.7 (3.9-35) | 3973.8 (876-7091.6) | 456.7 (98.3-808.6) | 0.77 (0.57-0.98) | 0.56 (0.32-0.79) |
| Togo | 127.6 (24.7-218.8) | 11.9 (2.3-20.8) | 3546 (716.9-6026.3) | 269.6 (52.6-462.2) | 392.7 (81.6-709.3) | 12.1 (2.4-21.6) | 11228.2 (2436.7-20269.5) | 271.3 (56.2-490.1) | -0.12 (-0.37-0.13) | -0.14 (-0.4-0.13) |
| Tokelau | 0.1 (0-0.1) | 5.9 (1-11.8) | 1.8 (0.3-3.5) | 140.9 (24.3-281.5) | 0 (0-0.1) | 2.2 (0.4-4.6) | 0.7 (0.1-1.6) | 51.6 (9.4-110) | -3.56 (-3.86--3.27) | -3.67 (-3.98--3.37) |
| Tonga | 1.7 (0.3-3.4) | 3.4 (0.6-6.9) | 47.5 (9.6-93.7) | 82.9 (16.6-164.7) | 1.5 (0.3-3.2) | 1.9 (0.3-4.1) | 36.7 (6.8-79.5) | 44.7 (8.2-97.6) | -1.29 (-1.5--1.09) | -1.52 (-1.73--1.31) |
| Trinidad and Tobago | 156.5 (29.7-269.7) | 21.1 (4-36.4) | 4029.7 (796.1-6862.8) | 479.7 (93.9-824.3) | 112.8 (20.3-210.2) | 6.1 (1.1-11.4) | 2712.5 (510.2-5004.7) | 145.4 (27.5-267.2) | -5.11 (-5.53--4.69) | -5.03 (-5.49--4.58) |
| Tunisia | 1030.5 (193.6-1775.8) | 24.4 (4.6-42.7) | 26836.3 (5230.2-45157) | 532.7 (102.7-907.5) | 1731.3 (314-3254.6) | 14.3 (2.6-26.9) | 39475.2 (7710-72856.5) | 300.2 (58.1-552) | -2.12 (-2.24--1.99) | -2.26 (-2.39--2.13) |
| Turkey | 6938.6 (1488.4-11955.5) | 21.7 (4.5-38.1) | 189743.9 (42442.1-317927.2) | 513.8 (112.6-868.6) | 7149.6 (1482.8-12942.5) | 8.4 (1.7-15.2) | 151470.4 (32525.5-269045.8) | 167.3 (35.8-296.5) | -3.16 (-3.46--2.86) | -3.93 (-4.19--3.68) |
| Turkmenistan | 752.4 (149.6-1280.5) | 45.4 (8.9-77.7) | 19090.9 (3914.5-31543.2) | 991.5 (200.1-1662.5) | 1035.5 (208.1-1848.5) | 29.8 (5.8-53.6) | 25185.9 (5459.8-44926.9) | 626.4 (130.5-1110) | -2.52 (-3.11--1.92) | -2.66 (-3.32--2) |
| Tuvalu | 0.8 (0.2-1.5) | 13.8 (2.8-25.2) | 25.2 (5.4-46.4) | 357.6 (76.1-652.1) | 0.7 (0.1-1.3) | 7.1 (1.4-14.3) | 18.7 (3.6-36.2) | 177.9 (34.7-342) | -1.57 (-1.77--1.37) | -1.65 (-1.87--1.44) |
| Uganda | 265.9 (51.7-493.7) | 4.8 (0.9-9) | 7356 (1493.4-13366.4) | 109.1 (21.7-200.8) | 428.4 (75.5-814.2) | 3.3 (0.6-6.3) | 12342.2 (2250.8-23476.9) | 74.5 (13.3-141.9) | -2.12 (-2.6--1.64) | -2.3 (-2.81--1.78) |
| Ukraine | 16547.6 (2974.3-29687.6) | 25.9 (4.6-46) | 333739.6 (62498.5-593865.4) | 493.3 (92.6-874.7) | 21600.5 (4068.9-39917.1) | 27.5 (5.2-50.4) | 394089.8 (77146.7-721986.5) | 519.7 (102.8-947.7) | -0.88 (-1.66--0.09) | -0.96 (-1.76--0.15) |
| United Arab Emirates | 55 (10.7-103.6) | 13.5 (2.3-25.5) | 1859.2 (374.9-3414) | 301.2 (54.3-561.1) | 201.4 (38.4-356.9) | 9.1 (1.6-16.8) | 7177.1 (1388.1-12512.8) | 168.1 (30.8-309.9) | 0.42 (-0.12-0.96) | -0.64 (-1.06--0.22) |
| United Kingdom | 9705.9 (1871.7-17448.5) | 10.6 (2.1-18.9) | 190703.8 (37450-338298.2) | 223 (45-393.6) | 2766.6 (557.6-5436.2) | 1.9 (0.4-3.7) | 48534.9 (9828.2-91086.5) | 39.7 (8.1-73.7) | -5.89 (-6.14--5.63) | -5.93 (-6.21--5.65) |
| United Republic of Tanzania | 490.7 (100.3-883) | 5 (1-9.1) | 14134 (2975.6-25113.2) | 121.1 (24.8-215.2) | 1421.7 (265.1-2639.2) | 6 (1.1-11.3) | 40780.8 (8100.8-74976) | 141.8 (26.6-261.1) | 0.31 (-0.03-0.65) | 0.22 (-0.14-0.57) |
| United States of America | 41748.4 (7683.3-74730.8) | 12.9 (2.4-22.9) | 814774.9 (153882.8-1414244.4) | 264.5 (50.5-457.5) | 36886.2 (6932.3-66672) | 6.2 (1.2-11) | 737234.7 (144967.3-1282830.8) | 137.2 (27.4-234.5) | -2.67 (-2.87--2.48) | -2.35 (-2.53--2.17) |
| United States Virgin Islands | 7.8 (1.4-14.1) | 11.4 (2-20.6) | 200.5 (38.1-357.8) | 235.4 (43-422.4) | 4.4 (0.7-8.9) | 2.7 (0.4-5.5) | 83.9 (13.7-163.8) | 54.6 (9.1-103.8) | -4.6 (-5.27--3.92) | -4.73 (-5.48--3.97) |
| Uruguay | 691.9 (136.1-1177.1) | 18.2 (3.6-31) | 14817 (2999.1-24768.3) | 396.6 (81-660.4) | 347.4 (61.5-611.1) | 5.8 (1-10) | 6671.5 (1240.2-11398.5) | 128.1 (24.5-215.3) | -3.77 (-3.93--3.61) | -3.74 (-3.9--3.58) |
| Uzbekistan | 5020.5 (1060-8321.7) | 46.6 (9.7-77.8) | 120473.1 (26861.4-193076.5) | 1029.6 (225.8-1663.9) | 10048.8 (1951-17262) | 46.3 (8.8-80.2) | 250266.4 (50662.5-419223.9) | 957 (188.7-1634.9) | 0 (-0.4-0.4) | -0.3 (-0.72-0.12) |
| Vanuatu | 7 (1.4-13.2) | 12 (2.2-23.1) | 235.8 (49.4-437.1) | 311.1 (61.6-585.1) | 19.6 (4.1-37.5) | 12.1 (2.4-23.1) | 646.9 (140.7-1219.9) | 314 (66.1-597.8) | -0.11 (-0.21-0) | -0.14 (-0.23--0.04) |
| Venezuela (Bolivarian Republic of) | 1152.7 (221.2-2018.2) | 12.6 (2.4-22.3) | 31194.6 (6295.6-53445.5) | 295.1 (58-508.7) | 3335 (629.4-6060.2) | 11.5 (2.1-20.9) | 81974.9 (16981.5-146164) | 270.1 (55.5-482) | -1.46 (-1.98--0.93) | -1.56 (-2.1--1.01) |
| Viet Nam | 2138.4 (410.9-3816.1) | 5.8 (1.1-10.4) | 54038 (11049.6-93992.6) | 133 (26.8-231.4) | 1326.9 (223.2-2682.7) | 1.6 (0.3-3.2) | 29630.9 (4970.3-59886.4) | 30.9 (5.2-61.9) | -3.86 (-4.07--3.65) | -4.37 (-4.58--4.17) |
| Yemen | 2029.2 (416.5-3600.9) | 44.3 (8.5-79.1) | 61854.3 (13500.1-110954.2) | 1120.5 (232.7-1981.8) | 4735 (1070.5-8465.4) | 36.4 (7.9-65.1) | 139023.6 (32654.6-242649.8) | 862.3 (196.2-1536.7) | -1.01 (-1.2--0.83) | -1.25 (-1.43--1.06) |
| Zambia | 126 (25.3-218.9) | 5 (1-8.6) | 3720.5 (787.6-6394.8) | 119.3 (24.4-204.9) | 376.4 (80.8-706.2) | 6.1 (1.2-11.4) | 11277.8 (2504.7-21216.4) | 140.7 (30.1-264.5) | 0.28 (-0.02-0.58) | 0.09 (-0.26-0.44) |
| Zimbabwe | 350.3 (73-607.4) | 11 (2.2-19.1) | 8847.3 (1903.8-15152.4) | 225.4 (47.4-389.5) | 999.8 (211.6-1712.4) | 17 (3.5-29.6) | 29198.6 (6481-50099.9) | 391.5 (83-667.2) | 2.14 (1.53-2.76) | 2.49 (1.8-3.19) |

Supplementary Table 1. Ischemic heart disease mellitus burden attributable to diet low in seafood omega-3 fatty acids in 1990 and 2021 and its temporal trends from 1990 to 2021 by nation.
